# The characteristics of effective technology-enabled dementia education: a systematic review and mixed research synthesis

**DOI:** 10.1186/s13643-021-01866-4

**Published:** 2022-02-23

**Authors:** Kevin Muirhead, Leah Macaden, Keith Smyth, Colin Chandler, Charlotte Clarke, Rob Polson, Chris O’Malley

**Affiliations:** 1 Department of Nursing & Midwifery, School of Health, Social Care & Life Sciences, University of the Highlands and Islands, Centre for Health Science, Old Perth Road, Inverness, IV2 3JH UK; 2 Learning and Teaching Academy, University of the Highlands and Islands, An Lòchran, Inverness Campus, Inverness, IV2 5NA UK; 3 School of Health in Social Science, University of Edinburgh, Buccleuch Place, Edinburgh, EH8 9LN UK; 4 Faculty of Social Sciences and Health, Durham University, Arthur Holmes Building, Lower Mountjoy, South Road, Durham, DH1 3LE UK; 5 Highland Health Sciences Library, Centre for Health Science, Old Perth Road, Inverness, IV2 3JH UK

**Keywords:** Dementia, Dementia education, Dementia training, Technology-enabled learning, Mixed research, Systematic review

## Abstract

**Background:**

Dementia education is required to address gaps in dementia-specific knowledge among health and social care practitioners amidst increasing dementia prevalence. Harnessing technology for dementia education may remove obstacles to traditional education and empower large communities of learners. This systematic review aimed to establish the technological and pedagogical characteristics associated with effective technology-enabled dementia education for health and social care practitioners.

**Methods:**

MEDLINE, PubMed, Web of Science, CINAHL, Scopus, PsycINFO, ERIC and OVID Nursing Database were searched from January 2005 until February 2020. Quantitative, qualitative and mixed methods studies were eligible for inclusion. Study quality was assessed with the Mixed Methods Appraisal Tool. Quantitative evidence was categorised based on Kirkpatrick’s Model. Qualitative data was synthesised thematically and integrated with quantitative findings before conclusions were drawn.

**Results:**

Twenty-one published papers were identified. Participants were acute, primary and long-term care practitioners, or were students in higher education. Most training was internet-based; CD-ROMs, simulations and tele-mentoring were also described. Technology-enabled dementia education was predominantly associated with positive effects on learning outcomes. Case-based instruction was the most frequently described instructional strategy and videos were common modes of information delivery. Qualitative themes emerged as *existing strengths and experience*; *knowledge gaps and uncertainty*; *developing core competence and expertise*; *involving relevant others*; and *optimising feasibility*.

**Discussion:**

Technology-enabled dementia education is likely to improve dementia knowledge, skills and attitudes among health and social care practitioners from multiple practice contexts. Confidence in the results from quantitative studies was undermined by multiple confounding factors that may be difficult to control in the educational research context. Convenience and flexibility are key benefits of technology-enabled instructive and simulated pedagogy that can support the application of theory into practice. More research is required to understand the role of online learning networks and provisions for equitable engagement. A future emphasis on organisational and environmental factors may elucidate the role of technology in ameliorating obstacles to traditional dementia education.

**Systematic review registration:**

PROSPERO (CRD42018115378)

**Supplementary Information:**

The online version contains supplementary material available at 10.1186/s13643-021-01866-4.

## Background

Dementia is a chronic and progressive syndrome in which there is disturbance of multiple higher cortical functions. Alzheimer’s Disease (AD), vascular dementia, dementia with Lewy bodies and frontotemporal dementia are common subtypes although boundaries are indistinct and mixed forms co-exist [1, 2]. The global prevalence of people living with dementia is approximately 50 million and it is predicted to rise to 152 million by 2050 [3]. Within the UK, 850,000 people (one in 14 adults over the age of 65) are estimated to be living with dementia and future prevalence is predicted to mirror global trends [4]. Despite reports of a decrease in age-specific incidence [5, 6], dementia remains a global public health priority [7].

UK dementia policy and strategies have been implemented to improve care for people living with dementia with key objectives that include improved diagnosis rates, post-diagnostic support and workforce development through staff education and training [8–13]. The need for an appropriately educated and dementia aware health and social care workforce responds to demographic transition and also concern about care quality for people with dementia [14]. This requirement permeates the entire health and social care spectrum—it is not limited to mental health professionals and designated dementia care settings [15]. In the UK, one in four hospital beds are occupied by someone living with dementia [4]; however, research suggests that there are gaps in dementia knowledge and skills among acute healthcare practitioners [16]. Hospital admission can present challenges for people with dementia who may struggle to adapt due to the disruptive effects of the acute care environment [17]. Inadequate staff training and knowledge around dementia care can result in unmet care needs and an increase in behavioural and non-cognitive symptoms—which practitioners perceive to be burdensome [18]. Among primary healthcare practitioners, more dementia education is required to address low rates of diagnosis, inappropriate specialist referral and sub-optimal patient management [19]. Gaps in dementia knowledge and skills can also exist among social care practitioners working in specialist dementia services including residential and nursing homes [20]. Inadequate dementia training among the long-term care workforce has been linked to substandard care and job dissatisfaction [21]. Effective dementia education that is embedded within pre-registration health and social care programmes will ensure that the future workforce can carry forward the appropriate knowledge, skills and attitudes that are required to work with people with dementia [22].

Technology-enabled learning has gained popularity due to low costs, high flexibility and reduced dependence on geographical boundaries [23]. It is increasingly being adopted in medical and healthcare educational contexts and may be as effective as traditional learning for knowledge and skills acquisition [24]. Convenience and flexibility are key benefits for health and social care practitioners (HSCP) who may experience challenges addressing professional development. Technology can efficiently remove many logistical barriers to traditional HSCP education and offers individualised and point-of-care learning for professionals from a wide range of practice settings [25]. Communication and collaboration are optimised by Web 2.0 technology [26, 27] which characterises the transformation of the static ‘read only’ Web 1.0 into a dynamic ‘read-and-write’ participatory media that has generated a new paradigm for teaching and learning by offering interconnectivity, interactivity, and content creation [28]. Web 2.0 tools include blogs, wikis and networking platforms that enable, and make visible, the social construction of new knowledge [29].

The COVID-19 pandemic resulted in an overwhelming transition to virtual teaching and learning as a preventative measure to contain the spread of the virus [30]. This represented a transformation and further advancement of digital healthcare education with new practices and principles evolving from a surge in uptake and new knowledge gained [31]. Healthcare educators who will harness the potential of technology for dementia education may contribute to a transformation of dementia education and empower large communities of learners. However, educational technology is not a panacea as learners may experience technical issues, have reduced social contact or have inadequate skills for learning with technology. There is also the requirement for knowledge translation into clinical practice. This is a complex phenomenon which can be influenced by the nature of the knowledge, the target audience, the expected outcomes and the instructional methods [32]. Dementia education has relevance to the entire health and social care workforce. HSCPs are key stakeholders who require training to develop the knowledge, skills, and attitudes required to meet the complexities of dementia care in practice. Kirkpatrick’s Four-Level Model provides a framework for the expected outcomes. These include learners’ *reactions* to the training; *learning gains* as knowledge, skills and attitudinal change; practice-based *behaviour change* following training; and the wider *results* due to the training [33]. The model has been applied effectively to the dementia education context [34–36]. An emphasis on instructional methods and pedagogy ensures that outcomes cannot be attributed to technology per se. In this way, the technology is considered *enabling* of dementia education [37].

Several reviews of dementia education have been conducted [22, 34–36, 38–49]; however, there has been limited focus on technology-enabled approaches to dementia education. In their comprehensive review of effective dementia education for the health and social care workforce, Surr et al. [35] highlighted that web-based training using *interactive* learning approaches were found to increase practitioner confidence, competence and self-efficacy. In general, active learning (e.g., using online multimedia methods) was considered to be more effective than passive approaches (e.g., watching an online video lecture). Online discussions were considered beneficial to learning; however, time demands and the need for specialist technical support suggested this to be a resource intensive form of study. Scerri et al. [34] suggested that e-learning may not always be feasible in healthcare settings due to limitations in participants’ time, internet access and digital competence. In contrast, evidence also suggests that the flexibility of dementia e-learning can be beneficial [35]; however, Surr and Gates [36] recommended that learners do not schedule time for their own e-learning due to difficulties in negotiating adequate time for learning—particularly in areas where there are significant work pressures and staff shortages. Scerbe et al. [41] was the only review that focussed exclusively on digital modes of dementia education and reported predominantly positive post-training effects on dementia knowledge, care strategies, communication skills, self-efficacy and attitudes among various healthcare practitioners.

Surr et al. [35] identified key features for *effective dementia education* for HSCPs which supports the application of approaches to professional development more broadly. Moehead et al. [50] determined key features associated with *effective web*-*based training* which were correlated with a web-based dementia training programme. Table 1 demonstrates effective dementia education features as they relate to key features of effective web-based learning—providing support for technology-enabled dementia education (TEDE). Features of effective dementia education require additional emphasis on training duration, theoretical determinants underpinning practice-based learning and provision of structured guidance for dementia care. Effective web-based training requires additional focus on cost-efficiency, accessibility and the reinforcement of learning. Healthcare education policy will benefit from a robust evidence-base from which practitioners can base their practice [51]. There is potentially more to be known about the effectiveness of TEDE and associated pedagogical characteristics within health and social care, and health science educational contexts. TEDE is a relatively new research area which will require cumulative and robust contemporary evidence that reflects the rapid pace of digital transformation.

**Table 1:** Features associated with effective dementia education & web-based learning

Effective dementia education features Surr et al. [35]	Effective web-based learning features Moehead et al. [50]
Relevant and realistic to the role and experience of learners rather than a one-size-fits-all approach	• Individualised and based on learner’s profile and background • Self-directed and self-paced • Flexible • Provides equitable engagement
Includes active participation	• Interactive • Multimodal
Ensures that experiential and simulation-based learning include adequate time for debriefing and discussion	• Nurtures critical thinking and reflection
Delivered by experienced trainers who can adapt training to the needs of each group	• Facilitated, access to instructor, or mentored • Flexible
Avoids reading written materials (paper or web-based) or in-service learning as the sole teaching method	• Multimodal • Interactive • Flexible
Includes active, small, or large group face-to-face learning either alone or in addition to another learning approach	• Interactive • Establishment of a learning community • Multimodal
Includes learning activities that support the application of training into practice	• Ability for translation into practice • Measures using questionnaires, feedback, and surveys of satisfaction

### Aim

This systematic review aimed to establish the technological and pedagogical characteristics associated with effective technology-enabled dementia education for health and social care practitioners. The research questions were:

- ▪ What are the methodological strengths and limitations of studies that evaluate technology-enabled dementia education programmes for health and social care practitioners?
- ▪ How do educational theories guide the design and development of technology-enabled dementia education programmes for health and social care practitioners?
- ▪ Can technology-enabled dementia education improve dementia knowledge, skills and care attitudes among health and social care practitioners?
- ▪ What pedagogical and technological characteristics are associated with technology-enabled dementia education for health and social care practitioners?
- ▪ What are the perceptions and experiences of technology-enabled dementia education among health and social care practitioners?

## Methods

The content of this systematic review was informed by the Preferred Reporting Items for Systematic Review and Meta-Analysis (PRISMA) guidelines and checklist [52] (Additional file 1).

### Protocol

The review protocol was registered on PROSPERO (CRD42018115378) and published [2].

### Criteria for including studies

Table 2 demonstrates the ‘PICOS’ criteria for including studies in the review.

**Table 2:** PICOS

Parameter	Inclusion criteria	Exclusion criteria
Participants	All HSCPs with, without, or working towards a professional qualification or registration participating in TEDE in any workplace or educational setting.	Informal (family) caregivers.
Intervention	Any technology-enabled approach to dementia education^a^ for HSCPs including single interventions, modules, and courses.	Decision support, DVD/video, and telephonic interventions.
Comparator	Studies involving comparator groups or no comparator groups.	**–**
Outcome	Primary outcome measures were satisfaction; knowledge, skills, and attitudes; behaviours; and results. Secondary outcomes included the educational theories informing TEDE; usability of TEDE; facilitators and barriers to TEDE.	**–**
Study design^b^	Randomised controlled trials and quasi-experimental studies. Quasi-experimental studies included pre- and post-test designs, control group designs (with or without dependent pre- and post-tests) and time-series designs. Mixed methods studies reporting robust qualitative data collection and analysis methods alongside experimental research methods^c^. Qualitative studies that described participant perceptions of TEDE characteristics/effects.	Descriptive studies and programme evaluations with narrative or survey data of participants’ general impressions of TEDE. Studies not published in the English language. Studies published before 2005^d^.

^a^ TEDE was defined for this review as: ‘a learning or teaching approach to dementia education that is fully or partially mediated by information communication technology’.

^b^ Study selection was intentionally broad to provide a comprehensive account of the various TEDE characteristics. This also recognised the diversity of educational research methods [53]

^c^ Experimental studies reporting additional narrative or survey evaluations of participants’ general impressions of TEDE were not considered to be mixed methods research and were classified as quantitative studies

^d^ The search was limited to 2005 to reflect technological progress since Web 2.0 [54]

### Search methods

Literature searches were carried out in MEDLINE (OVID interface), CINAHL Complete (EBSCO interface), ERIC (EBSCO interface), PsycINFO (EBSCO interface), PubMed, Web of Science Core Collection, OVID Nursing Database and SCOPUS from January 2005 until November 2018. Studies published before 2005 were not sought so that the review was based on contemporary evidence that reflects the rapid pace of technological progress and pedagogical opportunities since Web 2.0. Keywords included dementia (and subtypes), education and multiple terms for technological modes of education, learning or training. The search was updated in February 2020. The multi-database search strings are available (Additional file 2).

### Data collection and analysis

#### Selection of studies

The titles and abstracts of studies identified from the search were screened by one reviewer (KM). Two other reviewers (LM and CC) independently screened 10% of the titles and abstracts identified in the initial search—by each screening five percent. Second reviewer title and abstract screening was conducted with rigour. All studies identified in the initial search were ordered alphabetically and stratified into blocks of 10. Each block was then ordered numerically and each second reviewer was assigned to either odd or even numbered blocks. The second reviewers were then required to randomly identify one study per block for title and abstract screening. All reviewers used the same eligibility protocol and any conflicting decisions regarding eligibility were resolved though discussion without the need for third party arbitration. Second reviewer screening was used only at the title and abstract review stage; therefore, the full texts of potentially eligible studies were assessed by one reviewer (KM). Studies that did not satisfy the eligibility criteria following full text review were removed and issued with an exclusion rationale. Studies that were published in a foreign language were discarded as it was not possible to determine their eligibility for inclusion. The reference lists of all eligible studies were screened by one reviewer (KM) and studies that met the eligibility criteria were included.

#### Data extraction

Standardised quantitative and qualitative data extraction forms were developed for the review context. The data extracted included specific details about the study, participant characteristics, and the exposure of interest (TEDE) including the technological and pedagogical characteristics. Outcome data of significance to the review questions were also extracted. In quantitative studies, this involved extracting data relevant to the primary outcomes (i.e. data relevant to Kirkpatrick’s Model) and associated secondary outcomes. In qualitative studies, all data from the ‘findings’ and/or ‘results’ sections from primary studies were extracted to facilitate the subsequent coding that would precede the generation of themes. The data extraction forms also included evidence from the evaluation of methodological quality of primary studies. The forms were pilot tested before application and all data was extracted by one reviewer (KM). Sample data extraction forms are provided (Additional file 3).

#### Assessment of methodological quality

The Mixed Methods Appraisal Tool (MMAT) Version 2018 [55] was applied for methodological quality assessment. MMAT is a generic critical appraisal tool designed for systematic mixed studies reviews with specific categories for qualitative research (MMAT1), randomised controlled trials (RCTs)–(MMAT2), non-randomised studies (MMAT3) and quantitative descriptive studies (MMAT4). MMAT can also be used to appraise the overall quality of mixed methods studies (MMAT5). In the current review, MMAT was utilised for the independent assessment of quantitative and qualitative research methods. This approach recognised the binary distinction between quantitative and qualitative research and was considered optimal for subsequent data synthesis which would also employ a segregated approach. Each MMAT category has a specific quality criteria with three response options: ‘Yes’ means that the criteria was met, ‘No’ means that the criteria was not met and ‘Can’t tell’ means that there was not enough information in the paper to judge the criteria. One reviewer (KM) appraised the quality of the quantitative studies using MMAT2 and MMAT3. Second reviewers (LM and KS) appraised the quality of 19% of these studies with disagreements resolved through discussion. The quality of the qualitative evidence was appraised by one reviewer (KM) using MMAT1. The overall methodological quality within and across studies was based on proportions of ‘Yes’, ‘Can’t tell’ and ‘No’ judgements. All quality domains were included in the assessments (i.e. all quality domains were considered important) (Table 3).

**Table 3:** Approach to formulating summary assessments of methodological quality (across domains) within and across studies

Methodological quality	Interpretation	Within a study	Across studies
Low	Bias may seriously weaken confidence in the results.	The criteria was not met in one or more of the quality domains.	Most information was from low quality studies.
Moderate	There is a risk of bias that raises some doubt about the results.	The criteria was met, or there was not enough information to judge if the criteria was met for all quality domains.	Most information was from high or moderate quality studies.
High	Bias, if present, is unlikely to alter the results seriously.	The criteria was met in all the quality domains.	Most information was from high quality studies.

Adapted: [56, 57]

#### Data synthesis

Key study information was presented in a summary table including intervention characteristics and effects. MMAT scores were included so that intervention effects could be considered in relation to the methodological quality of studies. Thereafter, quantitative and qualitative data were synthesised independently [58]. Intervention effects were established from mean, median or percentage pre- to post-test increases or between group differences that favoured the TEDE. The findings were organised by primary outcomes with care settings in subgroups. Qualitative data was synthesised thematically. An inductive coding system was applied to small data segments of shared content from ‘findings’ or ‘results’ in primary studies. The initial codes were then grouped together into broader themes. Findings were reported in a narrative summary with representative participant quotations where relevant. The quantitative and qualitative syntheses were then combined. The quantitative findings provided evidence for the effectiveness of the TEDE interventions which was complimented, confirmed or refuted by the qualitative evidence. The qualitative data also provided a more comprehensive understanding of the essential characteristics of TEDE through the identification of additional theory. Specific descriptions of educational content, pedagogical characteristics, and educational theories described within the primary studies were not included in the combined synthesis and were reported separately.

## Results

### Results of the search

**Fig. 1:**
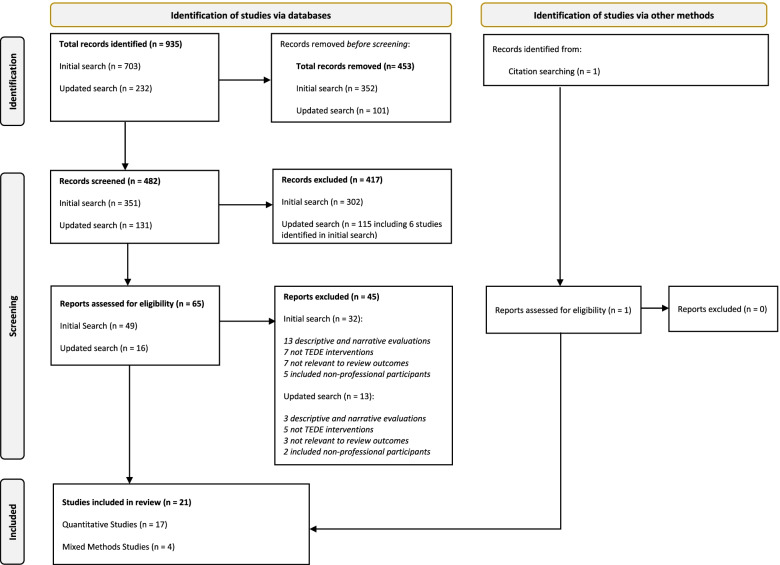
Prisma Diagram [59]

The total number of studies included in the review was 21. The initial search resulted in a total of 935 potentially eligible records. A total of 453 duplicate records were identified and removed, and the titles and abstracts of 482 remaining records were screened for relevance based on the eligibility criteria. From these, 417 records were considered to be ineligible and the full texts of 65 records were retained for full-text review. Forty-five records were excluded as they focused on descriptive and narrative-based evaluations of TEDE; were not TEDE interventions; were not relevant to the review outcomes; or included non-HSCPs (informal/family carers). The remaining 20 studies plus an additional study identified from the reference lists of eligible studies were included in the final synthesis (Fig. 1).

### Included studies

All the included studies reported on the effectiveness of TEDE using experimental methods. Four studies described robust mixed methods [60–63]. There were no standalone qualitative studies identified; however, one descriptive case study used experimental methods to establish learning outcomes [64]. This study was classified as quantitative research to satisfy relevant quality appraisal criteria and for appropriate positioning within the synthesis.

The 21 studies originated from nine countries. Ten studies were conducted in the USA. Australia, Canada and the UK produced two studies each. The remaining studies were from Brazil, Germany, Japan, Jordan and Taiwan. The oldest studies were published in 2006 [65, 66], and the most recent study was published in 2020 [67]. Most quantitative studies used a pre- and post-test design, or variant, including four randomised controlled trials [65, 68–70]. There were seven single group pre- and post-tests [60–62, 66, 71–73]; a single group pre- and post-test with follow up [64]; five equivalent/non-equivalent groups with pre- and post-tests [67, 74–77]; a three group pre- and post-test [63]; and a within subjects pre- and post-test [21]. The remaining studies included a nonrandomised study with control group [78]; and a one group repeated measure design [79].

Seven studies involved medical and nursing students in higher education [63, 66, 67, 70, 74, 75, 78], six included long-term care workers [21, 68, 71, 72, 77, 79], five studies involved primary care practitioners [60, 64, 65, 69, 76] and one study included hospital care workers [73]. One study involved participants from a variety of healthcare settings [61] and one combined participants from higher education and long-term care [62]. The number of participants varied between 421 GPs initially allocated to participate in an RCT [69] and 8 family physicians who participated in the descriptive case study [64].

All of the studies reported quantitative findings for either knowledge, skills, attitudes, behaviours or results. Several studies reported on multiple outcomes. Knowledge was assessed in all but four studies [65, 67, 77, 78]. Attitudinal change was assessed in seven studies [21, 60, 62, 63, 66, 68, 79]; as were skills [21, 66–68, 76, 78, 79]. Practitioner behaviours were considered in four studies [65, 70, 77, 79]. Broader results due to the training were explored in two studies [77, 79]. One study assessed the complete range of these primary outcomes [79]. Full details of the included studies are provided in the Characteristics of Included Studies (Additional file 4).

### Excluded studies

From 482 records identified during database searching, 417 were discarded following title and abstract screening. The full text of the remaining 65 studies was examined and 45 were judged to be ineligible based on the eligibility criteria. The excluded studies are presented with exclusion rationales (Additional file 5).

### Quality assessment of included studies

**Fig. 2:**
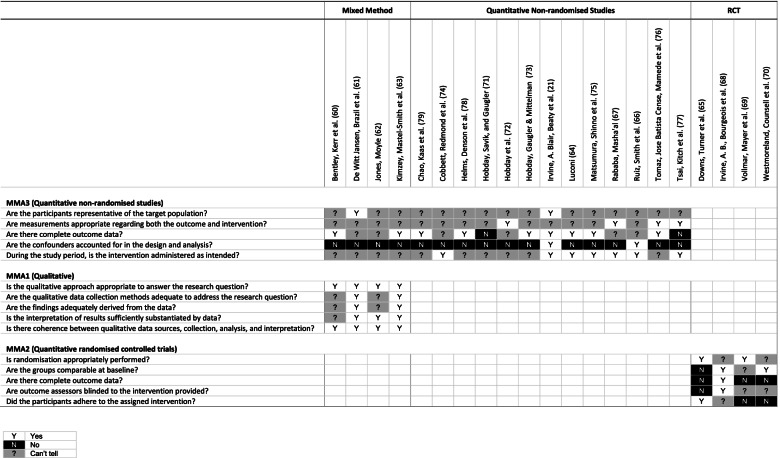
MMAT quality appraisal

Judgments on the quality of the individual studies using MMAT are shown in Fig. 2. Support for judgments are provided in Characteristics of Included Studies (Additional file 4).

### RCTs

**Fig. 3:**
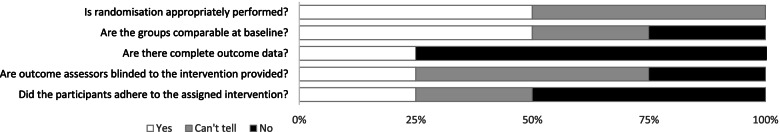
Methodological Quality Graph (RCTs)

RCTs were judged to be of low methodological quality overall. It was not clear if randomisation processes had been appropriately performed in two out of the four trials included in the review [68, 70]. Between group incomparability was identified in one of the trials [65], and it was not clear if between group similarities were significant in another [69]. An arbitrary threshold was applied for the assessment of outcome data. Acceptable dropout rates were considered to be < 20%, which negatively affected quality judgments in three trials [65, 69, 70]. One trial was unblinded [65], and it was not possible to tell if outcome assessors were blinded in two trials [69, 70]. Participant adherence may have been compromised in a trial using an unsupervised online dementia training [68]. Non-adherence was more obviously problematic in a trial where ‘non-users’ were identified [69], and in a trial that ended prematurely due to participant dissatisfaction [70]. Review authors’ judgements about each methodological quality item are presented as percentages across all included RCTs (Fig. 3).

### Quantitative non-randomised studies

**Fig. 4:**
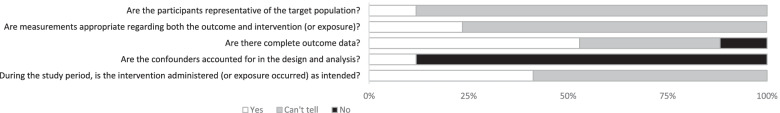
Methodological quality graph (quantitative non-randomised studies)

Overall, quantitative non-randomised studies were judged to be of low methodological quality. Several of the concerns identified were due to reporting limitations. It was frequently not possible to determine if participants were representative of target populations. Sampling methods were often not described, or there was insufficient information, in relation to sampling or target populations, for firm judgments. Convenience sampling methods were particularly problematic when assessing participant representativeness. Multiple outcome measures were often used within studies and it was frequently not possible to determine if these measures were appropriate. Limitations included inadequate reports of either validity or reliability, partial reporting of valid/reliable measures in studies using multiple measures, reports of validated measures that may not be reliable and vice versa, previously validated measures that were not validated in context, and measures with questionable reliability from sub-optimal alpha levels. An arbitrary threshold was applied to determine the completeness of the outcome data. Acceptable dropout rates were considered to be < 20%. It was frequently not possible to tell if the outcome data was complete. This was a common issue in pre- and post-tests due to insufficient reporting of participant numbers in either pre or post-tests. Two studies reported outcome data below the desired threshold [71, 77]. Interventions were assumed to have been administered as intended unless studies reported evidence to the contrary. ‘Can’t tell’ judgments were generally applied to studies that reported limitations to study processes, or where there were insufficient assurances of intervention controls including the location of participation. The main threat to study quality was from confounding factors which were either not described or accounted for in the study design or analysis. Time difference between pre and post-tests was a common source for potential confounding factors due to the possibility of maturation effects. Review authors’ judgements about each methodological quality item are presented as percentages across all included quantitative non-randomised studies (Fig. 4).

### Qualitative studies

**Fig. 5:**
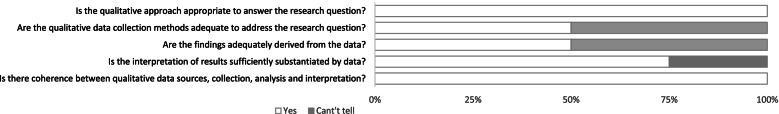
Methodological quality graph (qualitative studies)

Qualitative studies were judged to be of moderate methodological quality overall. Specific research methodologies were not reported; however, all studies described otherwise robust qualitative data collection and analysis methods. The form of the data collected and coding methods for data analysis were not described in two studies [60, 62]. Most studies provided representative quotations to justify themes that were identified in the data. Themes were less obvious in one study despite reporting a thematic approach to data analysis [60]. Coherence between qualitative data sources, collection, analysis and interpretation was judged to be satisfactory overall. Review authors’ judgements about each of the methodological quality item are presented as percentages across all included qualitative studies (Fig. 5).

### The characteristics and effectiveness of TEDE

A full summary of the included studies and intervention effects is shown in Table 4.

**Table 4:** Summary of included studies and intervention effects

Citation, Year, Country, Setting, Total Participants^**1,**^ Outcomes	Design, ***Intervention***, Intervention duration, Quality (MMAT)					Intervention characteristics	Comparison group(s)	*Continued* Results^**2**^
Bentley, Kerr et al. [60] 2019 Australia Primary Care IMG (*n* = 13), PN (*n* = 9) • Knowledge • Attitudes	Single Groups Pre- and Post-tests (in MMR) *Online interactive educational resource (recognising, diagnosing, and managing dementia in general practice).* 3-hour duration.					Online learning using video, assessment questions, and additional learning sources. Technological specifications not otherwise specified.	NA	**DKAS** *IMG* Pre-test Mean: 39.7 Post-test Mean: 43.7 *PN* Pre-test Mean: 34.1 Post-test Mean: 43.6 **GPACS-D** *IMG* Pre-test Mean: 11.1 Post-test Mean: 13.1 *PN* Pre-test Mean: 11.2 Post-test Mean: 13.0
	?	?	Y	N	?			
	**MMAT3**							
Chao, Kaas et al. [79] 2016 Taiwan Long Term Care Nurses (*n* = 107) • Knowledge • Attitudes • Skills • Behaviours • Results	One Group Repeated Measure. *Advanced Innovative Internet-Based Communication Education Program.* Total duration NS.					Blended learning approach using classroom / workshop with internet-based learning using videos, quizzes, online discussion and 360-degree reflective feedback. Technological specifications not otherwise specified.	NA	**CKS-C** Pre-test Mean: 62.42 (1.34)^3^, Sig: 0.00 Week 4 Mean: 69.79 (1.34)^3^, Sig: 0.00 Week 16 Mean: 73.25 (1.35)^3^, Sig:0.00 **CSAS-C** Pre-test Mean: 102.93 (0.80)^3^, Sig: 0.00 Week 4 Mean: 102.78 (0.80)^3^, Sig: 0.84 Week 16 Mean:104.12 (0.81)^3^, Sig: 0.11 **CCS** Pre-test Mean: 87.33 (1.02)^3^, Sig: 0.00 Week 4 Mean: 90.81 (1.02)^3^, Sig: 0.00 Week 16 Mean: 62.42 (1.34)^3^, Sig: 0.00 **PREAS**^**4**^ Pre-test Mean: 32.89 (0.43)^3^, Sig: 0.00 Week 4 Mean: 34.52 (0.43)^3^, Sig: 0.00 Week 16 Mean: 34.69 (0.43)^3^ Sig: 0.00 **RMBP-C**^**5**^ Pre-test Mean: 51.96 (1.44)^3^, Sig: 0.00 Week 4 Mean: 52.10 (1.44)^3^, Sig: 0.91 Week 16 Mean: 49.52 (1.44)^3^, Sig: 0.04 **CSDD-C**^**6**^ Pre-test Mean: 14.14 (0.62)^3^, Sig: 0.00 Week 4 Mean: 13.01 (0.62)^3^, Sig: 0.07 Week 16 Mean: 12.11 (0.63)^3^, Sig: 0.00
	?	?	Y	N	?			
	**MMAT3**							
Cobbett, Redmond et al. [74] 2016 Canada Higher Education Nursing Students (*n* = 129) • Knowledge • PRO	Non-equivalent control group pre- and post-test. *Alzheimer Disease and other Dementias Care Course: Adapted online course (ADODCC).* 8 online modules and 1 face-to-face. Module duration: 1 hour (with additional 2 hours of preparatory work).					Online course with modules that include readings and online specific activities such as discussions, wikis, personal journals, and quizzes. Technological specifications not otherwise specified.	Non-participation in ADODCC (*n* = 106)	**Pre- and post-test**^**7**^ *Intervention* Mean (Pre-Post): − 2.48 (1.56) *t* = − 7.61, *p* = 0.000 *Control* Mean (Pre-Post): − 0.46 (1.81) *t* = − 2.64, *p* = 0.010
	?	?	?	N	Y			
	MMAT3							
De Witt Jansen, Brazil et al. [61] 2018 UK Various^8^ Physician (*n* = 10), Nurses (*n* = 10) HCA (post intervention data not available) • Knowledge	Single groups pre- and post-test (in MMR). *Tele-mentoring to enhance assessment and management of pain in advanced dementia (based on Project ECHO model).* Clinics duration 1 hr 15 mins.					Tele-mentoring by experienced clinicians providing brief, focused didactic training. Learners present anonymised real patient cases for discussion. Clinics are digitally recorded. Participants can attend using Zoom videoconferencing technology.	NA	**ECHO Questionniares**^**9**^ *Physician* Pre-test Mean:41.4 (10.6) Post-test Mean: 55.8 (10.2) *p* = 0.014 *Nurse* Pre-test Mean:37.9 (6.5) Post-test Mean: 44.8 (7.0) *p* = 0.035
	Y	?	?	N	?			
	MMAT3							
Downs, Turner et al. [65] 2006 UK Primary Care GP Practices (*n* = 35) • Behaviours	Cluster randomised controlled before and after study. *Educational tutorial on CD-ROM.* Total duration NS.					CD-ROM using case analysis with emphasis on reflecting on knowledge and revisiting difficult and complex clinical problems. The tutorial was an ‘electronic book’ with indexing system and navigation using hypertext links. Technological specifications not otherwise specified.	Decision support software (*n* = 8) Practice based workshops(*n* = 9) Control–no intervention (*n* = 10)	**Detection Rates**^**10**^ *Intervention* Before: 43 (80); After: 11 (20) Difference^11^: 1.80, *p* = 0.18 *Workshop* Before: 47 (69); After: 21 (31) Difference^11^: 6.55, *p* = 0.02 *Decision Support* Before: 71 (70); After: 32 (30) Difference^11^: 7.31, *p* = 0.01 *Control* Before: 49(89); After: 6 (11)
	Y	N	N	N	Y			
	MMAT2							
Helms, Denson et al. [78] 2009 USA Higher Education Medical Students (*n* = 92) • Skills	Nonrandomised with control group. *E-module: Neurology and Dementia: Psychological Aspects of Care (with clerkship materials).* Total duration NS.					Hybrid learning including an e-module (intervention) in conjunction with didactic presentations of dementia as a clinical problem. E-modules included video segments hyperlinked to explanatory text and external resources, quiz, and module evaluation. E-module delivered using the ANGEL e-learning management system.	Neurology clerkship students not electing to participate in e-module (*n* = 26)	**OSCE (Performance)** Intervention Mean: 23.4 (3.3) Control Mean: 21.7 (2.9) *p* = 0.03 **OSCE (Clinical Note Overall)** Intervention Mean: 12.4 (2.4) Control group: 10.7 (1.9) *p* = 0.002
	?	?	Y	N	?			
	MMAT3							
Hobday, Savik, and Gaugler [71] 2010 USA Long Term Care Direct Care Workers (*n* = 34) • Knowledge • PRO	Single group pre- and post-test. *Internet-based Multimedia Education Program: Dementia training resource.* 3 prototype modules. Module duration 1 h.					Internet based training using text, real life videos, audio, and photographic content. Browser-based computer program using Adobe Flash. The program required a web browser (such as Internet Explorer or Netscape) and a Flash Player plug-in (downloadable as required).	NA	**Knowledge Inventory** Pre-test Mean: 10.6 (2.1) Post-test Mean: 12.6 (1.2) *t* = − 5.5, *p* < 0.001
	?	?	N	N	?			
	MMAT3							
Hobday et al. [72] 2010 USA Long Term Care Nurse Assistants (*n* = 40) • Knowledge • PRO	Single group pre- and post-test. *Internet-based, interactive, multimedia dementia educational program.* 4 modules / sessions. Module duration 1 hour.					Web-based learning using text, graphics, and video. Technological specifications not otherwise specified.	NA	**Dementia Care Knowledge** Pre-test Mean: 12.4 (1.9) Post-test Mean: 13.0 (2.0) *t* = − 2.6, *p* = 0.013
	?	Y	?	N	?			
	MMAT3							
Hobday, Gaugler & Mittelman [73] 2017 USA Hospital Nursing Assistants and Allied Hospital Workers (*n* = 25) • Knowledge • PRO	Single group pre- and post-test. *CARES Dementia-Friendly Hospital Program: Online dementia training program.* Total duration NS.					Online learning using audio-narrated text, graphics, video interviews, real life video scenarios, interactive text-entry, and case study scenarios asking learners to synthesize knowledge learned in real case scenarios. Learning completed on computers, tablets, or smartphones.	NA	**Dementia Care Knowledge** Pre-test percent: 82.2 (10.71) Post-test percent: 91.6 (6.08) *t* = 11.5, *p* < 0.001
	?	?	Y	N	?			
	MMAT3							
Irvine, A. B., Bourgeois et al. [68] 2007 USA Long Term Care Nurse aides (*n* = 62) • Knowledge • Attitudes • Skills • PRO	RCT (with pre- and post) tests. *Interactive multimedia program: Professional Dementia Care: Managing Aggression.* Total duration NS.					Internet training using storyboards incorporating graphics, video vignettes, and testimonials. After watching a video vignette (staff reaction to aggressive behaviour) learners chose correct answers from MCQs about the appropriateness of the response. Correct answers were reinforced, and incorrect answers were remediated with explanations. The learner then saw correct modelling of how to deal with the aggressive situation. Respondents who answered incorrectly were re-tested until correct responses were elicited. Text were written at 2^nd^-6^th^ grade reading level. Technological specifications not otherwise specified.	Control group did not participate in training program (*n* = 28)	**VST: Knowledge** *Intervention* Pre-test Mean: 1.62(0.82) Post-test Mean: 2.62(0.78) *Control* Pre-test Mean: 1.71 (1.05) Post-test Mean: 1.93(0.94) F17.02, *p* < 0.001 **Attitude**^**12**^ *Intervention* Pre-test Mean: 4.64(0.46) Post-test Mean: 5.08(0.44) *Control* Pre-test Mean: 4.61(0.42) Post-test Mean: 4.53 (0.32) F37.15, *p* < 0.001 **Behavioural Intentions**^**13**^ *Intervention* Pre-test Mean: 5.31(1.15) Post-test Mean: 6.17(0.96) *Control group* Pre-test Mean: 5.13(1.07) Post-test Mean: 5.39(1.19) F11.54, *p* < 0.001 **VST: Self-efficacy** *Intervention* Pre-test Mean: 3.15(0.80) Post-test Mean: 4.10(0.78) *Control* Pre-test Mean: 2.95(0.77) Post-test Mean: 3.25 (0.86) F26.55, *p* = 0.001 **Self-efficacy**^**14**^ *Intervention* Pre-test Mean: 4.92(1.13) Post-test Mean: 6.07(0.92) *Control* Pre-test Mean: 4.68(1.19) Post-test Mean: 4.97(1.26) F30.37, *p* < 0.001
	?	Y	Y	Y	?			
	MMAT2							
Irvine, A. Blair, Beaty et al. [21] 2013 USA Long Term Care Non-direct care staff including Nurses (*n* = 25) • Knowledge • Attitudes • Skills • PRO	Within-subjects pre- and post-tests. *Internet dementia-training program.* 5 modules. 2 hours to complete all modules.					Internet training using video-modelling vignettes, right-way and wrong-way exemplars, testimonials, and narration. Text written at a sixth-grade reading level. Technological specifications not otherwise specified.	NA	**VST: Knowledge** Pre-test Mean: 1.9(0.9) Post-test Mean: 2.3(0.6) *t* = 2.53, *p* = 0.018 **Attitudes** Pre-test Mean: 5.7(0.6) Post-test Mean: 6.0(0.5) *t* = 3.05, *p* = 0.005 **Behavioural intentions**^**15**^ Pre-test Mean: 5.5(0.7) Post-test Mean: 5.9(0.8) *t* = 2.02, *p* = 0.055 **VST: Self-efficacy** Pre-test Mean: 3.6(0.7) Post-test Mean: 3.8(0.5) *t* = 1.74, *p* = 0.094 **Self-efficacy**^**16**^ Pre-test Mean: 5.9(0.6) Post-test Mean: 5.9(0.8) *t* = 0.27, *p* = 0.787
	Y	?	Y	Y	Y			
	MMAT3							
Jones, Moyle [62] 2016 Australia Long Term Care Nurses, Care Workers, and Students (*n* = 42) • Knowledge • Attitudes	Single group pre- and post-tests (in MMR). *Online self-directed eLearning education intervention (based on the sexualities and dementia education resource for health* *professionals).* 4 modules. 4 hours to complete all modules.					E-learning (online) using case studies, activities, and resources. Technological specifications not otherwise specified.	NA	**ASKAS: Knowledge**^**17**^ Pre-test: Mean: 57.57 (15.06) Post-test Mean: 51.0 (8.56) *Z* = − 2.82, *p* = 0.005 **ASKAS: Attitude**^**18**^ Pre-test Mean 48.76(16.51) Post-test Mean 41.10(11.97) *Z* = − 2.57, *p* = 0.01 **SAID**^**18**^ Pre-test Mean 41.90 (10.88) Post-test Mean 37.38(7.48) *Z* = − 3.14, *p* = 0.002
	?	?	?	N	?			
	MMAT3							
Kimzey, Mastel-Smith et al. [63] 2016 USA Higher Education Nursing Students (*n* = 94)^19^ • Knowledge • Attitudes	Three groups pre- and post-tests (in MMR). *Alzheimer’s disease online module* Total duration NS.					Online learning. Characteristics not otherwise specified.	Experiential learning (*n* = 33) Control group - no dementia-specific intervention (*n* = 30)	**ADKS** *Intervention* Pre-test Mean:24.50 Post-test Mean: 24.75 *Experiential* Pre-test Mean: 23.42* Post-test Mean:25.58* *Control* Pre-test Mean: 23.43 Post-test mean: 23.37 *p ≤ 0.05 **DAS** *Intervention* Pre-test Mean: 108.03 Post-test Mean: 107.52 *Experiential* Pre-test Mean: 106.42* Post-test Mean: 118.56* *Control* Pre-test Mean: 110.17 Post-test Mean: 111.77 *p ≤ 0.05
	?	?	Y	N	?			
	MMAT3							
Luconi [64] 2008 Canada Primary Care Family Physicians (*n* = 8) • Knowledge • PRO	Single group pre- and post-test (with follow up). *Early Alzheimer’s Disease Program: Web-based Continuing Medical Education Program.* 8 modules completed over 6 months.					Web-based resource using mini-lectures and a case study approach. Individual and collaborative activities include asynchronous discussions, and hypertext links to resources. Designed and implemented on Web CT learning system. Basic hardware and software required are computer with internet access, a modem (56K or higher) or cable, and a web browser (Explorer 4 and up, or Netscape 6).	NA	**MCQ**^**20**^ Mean (Pre-Post): − 4.16(2.10) *t* = − 5.602, p0.001 Mean (Pre-FU): − 4.58(2.81) *t* = − 4.622, *p* = 0.002 Mean (Post-FU): − 0.43(1.68) *t* = − 0.716, p0.497 **Clinical Cases**^**20**^ Mean (Pre-Post): − 1.13(4.76) *t* = − 0.668, *p* = 0.526 Mean (Pre-FU): 0.50(5.53) *t* = 0.256, *p* = 0.805 Mean (Post-FU): 1.63(4.98) *t* = 0.922, *p* = 0.387
	?	?	Y	N	Y			
	MMAT3							
Matsumura, Shinno et al. [75] 2018 Japan Higher Education Medical Students (*n* = 79) • Satisfaction • Knowledge	Equivalent control group pre- and post-test. Clinical simulator with virtual patients (with conventional learning). Simulator duration 0.75 hours.					Simulated learning in practice concept with various virtual patients with dementia. Case study approach. Responses to patient questions using multiple choice approach, ordering diagnostic tests, giving prescriptions etc. with immediate virtual feedback or human interaction where necessary. Simulator can run in an internet browser or as a standalone system.	Conventional learning (*n* = 43)	**ARCS**^**21, 22**^ Pre-test Mean: 27.42(3.61) Post-test Mean: 29.56(3.88) *t* = 2.894, *p* = 0.007 **Knowledge Test** *Pre-test* Intervention Mean: 8.17(3.39) Control Mean: 8.42(4.03) *t* = 0.297, *p* = 0.767 *Post-test* Intervention Mean: 18.08(4.35) Control Mean: 15.51(4.32) *t* = 2.627, *p* = 0.01
	?	?	Y	N	Y			
	MMAT3							
Rababa, Masha'al [67] 2020 Jordan Higher Education Nursing Students (*n* = 104) • Skills • PRO	Equivalent control group pre- and post-test. *Computer-based BPS for pain management in people with dementia (and presentations and discussions using case scenarios / vignettes).* 6 training sessions of 1-hour duration.					Computer-based BPS. BPS is an interactive learning method using case scenarios that guide the learner though a step-by-step decision-making process. BPS gives learners the opportunity to make decisions according to their level of skills and knowledge and get feedback immediately which help them to demonstrate critical thinking skills in a safe and supported environment before dealing with complex and real-life case scenarios.	Traditional learning and vignettes (*n* = 52)	**CTSAS** *Intervention* Pre-test Mean: 337.67(13.44) Post-test mean: 660.52(10.57) *p* = <0.001 *Control* Pre-test Mean: 309.77 (15.11) Post-test mean: 499.64(14.78) *p* = 0.970
	?	Y	?	N	Y			
	MMAT3							
Ruiz, Smith et al. [66] 2006 USA Higher Education Nursing Students (*n* = 38) • Satisfaction • Knowledge • Attitudes • Skills	Single group pre- and post-tests. *Multimedia training CD-ROM: Alzheimer’s and other Dementias.* 7 modules. 20-30 minutes per module.					Computerised learning using text, animations, video, audio, and interactive exercises. CD-ROM for use on individual computers.	NA	**Knowledge Test** Pre-test Mean: 0.72 (0.02)^3^ Post-test Mean: 0.80 (0.02)^3^ *t* = 5.425, *p* < 0.001 **Attitude test** Pre-test Mean: 3.8 (0.15)^3^ Post-test Mean: 4.4 (0.13)^3^ *t* = 3.586, *p* < 0.001 **Self-efficacy**^**23**^ Pre-test Mean: 4.0 Post-test Mean: 4.7 *p* < 0.001
	?	?	?	Y	Y			
	MMAT3							
Tomaz, Jose Batista Cisne, Mamede et al. [76] 2015 Brazil Primary Care Family Physicians (*n* = 50) • Knowledge • Skills	Equivalent control group pre- and post-test. *Online PBL: Clinical Approach for Elderly with Dementia.* 120 hours (100 hours distance and 20 face-to-face) over 12 weeks.					Online PBL (with blended approach) involving virtual tutorial groups supervised by a facilitator using asynchronous virtual forums and synchronous chat. Online learning tools included video-lectures, CD-ROM, and texts. Online learning provided in learning management system (MOODLE).	Non-participation in online PBL (*n* = 25)	**Knowledge Test** *Intervention* Pre-test Mean: 6.38(0.96) Post-test Mean: 7.75(0.95) *Control* Pre-test Mean: 6.87(1.28) Post-test Mean: 7.15(0.77) F=17.98, *p* < 0.001 **MMSE** *Intervention* Pre-test Mean: 6.31(1.23) Post-test Mean: 8.22(0.60) *Control* Pre-test Mean: 5.93(1.12) Post-test Mean: 6.07(0.98) F=63.47, *p* < 0.001 **DD** *Intervention* Pre-test Mean: 5.31(1.71) Post-test Mean: 8.70(0.92) *Control* Pre-test Mean: 5.25(2.38) Post-test Mean: 5.66(2.59) *F* = 43.98, *p* < 0.001
	?	Y	Y	N	?			
	MMAT3							
Tsai, Kitch et al. [77] 2018 USA Long Term Care Nursing Assistants and Residents (*n* = 9 dyads) • Behaviours • Results • PRO	Equivalent control group pre- and post-test. *Computer-based simulation (level of dressing assistance for people with dementia) and face-to-face training module.* Simulator practice duration 2 hours.					Simulated learning using videos of an elder actor who does not perform dressing activities until the correct level of assistance is offered (selected). If the user selects inappropriate assistance, the elder responds with noncompliance or agitation. The goal is to minimise agitation, maximise independence, and enable dressing with minimal decision-making delays. Computer-based simulation on tablet device.	Face-to-face training module (*n* = 3)	**LOA / BDPS**^**24, 25**^ *Intervention* Median (Post-Pre): 33% *Control* Median (Post-Pre): 14.3% *p* = 0.42 **BDPS**^**26**^ *Intervention* Median (Post-Pre): -1.87 *Control* Median (Post-Pre): -0.28 *p* = 0.38
	?	Y	N	N	Y			
	MMAT3							
Vollmar, Mayer et al. [69] 2010 Germany Primary Care General Practitioners (*n* = 166 at T_1_ and *n* = 97 at T_2_) • Knowledge	Cluster randomised trial. *Blended learning (presentation of a dementia guideline in online modules and a structured discussion during a QC meeting).* The estimated average activity duration was 83 (15 to 200) minutes.					Blended learning approach at GP quality circles using online learning with interactive case studies, tests, and printed material. Technological specifications not otherwise specified.	Traditional lecture (and discussion in QC meeting) T_1_ (*n* = 82) T_2_ (*n* = 51)	**Knowledge Test (Dementia Management)**^**27**^ *T*_*1*_ *(T*_*1*_*-T*_*0*_*)* Intervention Mean: 3.67 Comparator Mean: 3.60 *t* = 0.15, *p* = 0.881 *T*_*2*_ *(T*_*2*_*-T*_*0*_*)* Intervention Mean: 2.39 Comparator Mean: 2.00 *t* = 0.636, *p* = 0.526
	Y	?	N	?	N			
	MMAT2							
Westmoreland, Counsell et al. [70] 2010 USA Higher Education Medical Residents (*n* = 77)^28^ • Knowledge • Behaviours	RCT. *Dementia education modules within a web-based geriatrics training program.* Total Duration NS.					Web-based training using case-based instruction. Modules were textual, with pictorial content. Video streaming was included to demonstrated how to administer the MMSE. Web-based learning on ‘A New Global Environment for Learning’ (ANGEL) curriculum repository.	Paper-based learning (*n* = 42)^28^	**Knowledge Test** *Intervention* Mean (Post-Pre): 20.0(16.6) *Comparator* Mean (Post-Pre): − 7.5(14.5) *p* < 0.001 **Encounter Checklist**^**29**^ Intervention Score: 56(15) Comparator Score: 56(18) *p* = 0.93 **Chart Abstraction**^**29**^ Intervention Score:41(12) Comparator Score: 33(13) *p* = 0.03 **EOES**^**29**^ Intervention Score: 84(15) Comparator Score: 79(20) *p* = 0.33
	?	Y	N	?	N			
	MMAT2							

*ADKS* Alzheimer’s Disease Knowledge Scale, *ARCS* Attention, Relevance, Confidence, and Satisfaction Motivational Model, *ASKAS* Ageing Sexual Knowledge and Attitudes Scale, *BDPS* Beck Dressing Performance Scale, *BPS* Branching path simulations, *CCS* Communication Competency Scale, *CKS-C* Communication Knowledge Scale–Chinese version, *CSAS-C* Communications Skills Attitudes Scale – Chinese version, *CSDD-C* Cornell Scale for Depression in Dementia–Chinese version, *CTSAS* Critical Thinking Self-Assessment Scale, *DAS* Dementia Attitudes Scale, *DD* Differential diagnosis, *DKAS* Dementia Knowledge Assessment Scale, *EOES* Electronic Order Entry Score, *GPACS-D* Confidence and Attitudes Towards Dementia Scale, *HCA* Healthcare assistant, *IMG* International medical graduates, *LOA* Level of assistance, *MCQ* Multiple choice questions, *MMR* Mixed methods research study, *MMSE* Mini-Mental State Examination, *NA* Not applicable, *NS* Not specified, *OSCE* Objective structured clinical examination, *PBL* Problem-based learning, *PN* Practice nurses, *PREAS* Patients Receptive and Expressive Ability Assessment Scale, *PRO* Participant reported outcomes, *QC* Quality Circle, *RCT* Randomised controlled trial, *RMBP-C* Revised Memory and Behaviour Problems Checklist – Chinese version, *SAID* Staff Attitudes about Intimacy and Dementia Survey, *VST* Video situation tests

Behavioural interventions classified as ‘attitudes’ as according to the Theory of Reasoned Action, attitudes are a direct determinant of behavioural intentions [80]. Self-efficacy classified as ‘skills’ as perceived self-efficacy is concerned with judgments of how well one can execute courses of action required to deal with prospective situations [81]. Self-efficacy has received similar classification in previous TEDE research [66]

^1^ Total participants (subtract number of comparator group participants from total participants for number of intervention group participants)

^2^ All results are presented as Mean (standard deviation) unless otherwise indicated

^3^ Mean (standard error)

^4^ Measured frequency of assessments

^5^ Measured memory and behavioural problems of people with dementia where higher scores indicate an increased frequency of problems

^6^ Measured depressive symptoms in people with dementia where higher scores indicate more depressive symptoms

^7^ Measured comprehension, application, and critical thinking

^8^ Primary and secondary care, nursing home, and hospice

^9^ Measured knowledge and self-efficacy

^10^ Number (percentage) of patients diagnosed with dementia. Concordance with dementia guidelines not included.

^11^ Wald test; comparison with control

^12^ Measured attitudes towards the importance of specific behavioural responses to aggressive situations

^13^ Measured participant’s intention to perform specific behaviours when dealing with aggressive situation

^14^ Measured confidence to perform specific behavioural responses to aggressive resident behaviours

^15^ Measured participants intention to perform specific behaviours in the next week

^16^ Measured confidence to respond as recommended to program-specific items

^17^ Lower scores indicated higher knowledge level

^18^ Lower scores indicated more permissive attitudes

^19^ Three outlier cases were removed from intervention group (AD module) in the ADKS due to negatively skewed data

^20^ Tests knowledge and skills related to the diagnosis, treatment, and management of early AD

^21^ Pre- and post-test results for experimental group only

^22^ Satisfaction only reported

^23^ Measured confidence in being able to provide dementia care

^24^ Use of appropriate level of assistance prompts based on subtask categories from BDPS

^25^ Higher scores indicate more appropriate levels of assistance given

^26^ Scores on various degrees of dependence for BDPS subtasks where higher scores indicate more dependency

^27^ Knowledge test completed at T_0_, T_1_, and T_2_. T_1_ is at 9 weeks after T_0_ and T_2_ is about 4 months after T_1_

^28^ Number reflects participants in knowledge test with variability in participant numbers for the other outcomes

^29^ Results are shown as score (percentage)

### Theories informing TEDE

Educational theories were not always reported. Chao et al. [79] described the application of principles from Knowles’ Adult Learning Theory to guide the development of their internet-based communication education programme. Accordingly, the authors acknowledged that ‘personal experience in life fosters a desire to understand or a need to perform a job more effectively, thus inspiring a higher motivation to learn’. Downs et al. [65] reported that the educational interventions included in their study reflected ‘different approaches to adult learning’. Hobday et al. [73] cited sources for incorporating effective adult learning principles into their online dementia modules and provided additional evidence of theory-based interactive design principles.

Cognitive constructivism and social constructivism were described in a comprehensive account of learning theories provided in the context of an online dementia education programme for rural physicians [64]. Cognitive constructivism was described as being ‘orientated towards the understanding of individual knowledge construction’ where learning is ‘an active, constructive, cumulative, and goal-oriented process’. Social constructivism, on the other hand, was defined as ‘the interdependence of social and individual processes for the co-construction of knowledge’ whereby learners ‘actively co-construct with others and the self’. The Four-Stage Theory of Physician Learning was described as having a basis in social constructivism and was included in a more focused theoretical framework for physician learning. Despite problem-based learning being referred to as a small group activity, Tomaz et al. [76] described this as simply being a ‘constructivist educational approach’ with influence from cognitive psychology. De Witt Jansen et al*.* [61] described ‘communities of learners’ in the context of their highly interactive tele-mentoring intervention. The Community of Practice Theory was cited to emphasise importance of ‘learning through continuous participation in a collaborative community consisting of peer learners and expert individuals’.

Rababa and Masha'al [67] explained that branching path simulation was informed by analytic decision-making learning theories and principles of behaviourism and cognitivism. Kimzey [63] discussed Kolb's Experiential Learning Theory which served as a guide for a dementia experiential learning intervention that was compared with TEDE. Tsai et al. [77] discussed Bandura’s Social Learning Theory and suggested that ‘behaviour can be learned through modelling and observation’ which supported their novel simulated training approach to reinforce appropriate caregiving techniques for optimal independence among long term care residents.

### The effectiveness of TEDE

Intervention effects were pre- to post-test increases or between group differences favouring TEDE. The first time point was used where studies included follow-up or time series data. Effectiveness was based on a positive direction of effect alone as reliance on statistically significant findings can result in limitations if underpowered studies (that do not report significant effects) are discarded [82]. A summary of intervention effects is shown in Table 5.

**Table 5:** Summary of intervention effects

Setting	Intervention	Knowledge	Skills	Attitudes	Behaviours	Results	Citation
Long-term care	Online learning	e^*^	–	e^*^	–	–	Jones, Moyle [62]
	Online learning^1^	e^*^	e^*^	n	e^*^	p	Chao, Keas et al. [79]
	Online learning	e^*^	–	–	–	–	Hobday, Savik, and Gaugler [71]
	Online learning	e^*^	–	–	–	–	Hobday et al. [72]
	Online learning	e^*^	e^*^	e^*^	–	–	Irvine, A. B., Bourgeois et al. [68]
	Online learning	e^*, 2^	p^2^	e^2^	–	–	Irvine, A. Blair, Beaty et al. [21]
	Simulation (video)	–	–	–	e	e	Tsai, Kitch et al. [77]
Primary care	CD-ROM	–	–	–	e^3^	–	Downs, Turner et al. [65]
	Online learning	e	–	e	–	–	Bentley, Kerr et al. [60]
	Online learning	e^*, 4^	–	–	–	–	Luconi [64]
	Online learning^1^	e^*^	e^*^	–	–	–	Tomaz, Jose Batista Cense et al. [76]
	Online learning^1^	e	–	–	–	–	Vollmar, Mayer et al. [69]
Hospital	Online learning	e^*^	–	–	–	–	Hobday, Gaugler & Mittelman [73]
Various	Tele-mentoring (online)	e^*^	–	–	–	–	De Witt Jansen, Brazil et al. [61]
Higher Education—Nursing	CD-ROM	e^*^	e^*^	e^*^	–	–	Ruiz, Smith et al. [66]
	Online learning^1^	e^*^	–	–	–	–	Cobbett, Redmond et al. [74]
	Online learning	e	–	n	–	–	Kimzey, Mastel-Smith et al. [63]
	Simulation (branch path)	–	e^*^	–	–	–	Rababa, Masha'al [67]
Higher Education—Medical	Online learning	–	e^*^	–	–	–	Helms, Denson et al. [78]
	Online learning	e^*^	–	–	p	–	Westmoreland, Counsels et al. [70]
	Simulation (clinic)	e^*^	–	–	–	–	Matsumura, Shinno et al. [75]

e (effective based on direct of effect); e^*^ (effective based on direction of effect and statistical significance where *p* < 0.05); n (not effective based on direction of effect); p (partial effectiveness based on direction of effect from multiple outcome measures)

^1^ Includes a blended learning approach

^2^ The findings relate to nurses only

^3^ Compared to control group for detection rates

^4^ The findings are for MCQ test only

TEDE was associated with mostly positive effects across the primary outcomes and in each practice setting. One study did not demonstrate positive attitudinal change among long term care practitioners—although some effects were noted over time [79]. In addition, nursing students’ attitudes did not improve following an online module; however, attitudinal change was observed in students who participated in an experiential learning arm of this study [63].

It is noteworthy that a greater proportion of non-significant findings were established at higher levels of Kirkpatrick’s model (i.e. behaviours and results). There was limited evidence to support practitioner learning gains as determinants of these broader outcomes. Satisfaction with TEDE was not reported in the summary of intervention effects as this outcome was rarely assessed using experimental methods.

### The characteristics of effective TEDE

#### Instructional and design features

All interventions were effective on at least one of the primary outcomes. Each study was screened for descriptions of intervention characteristics and the main pedagogical characteristics were noted. Online learning was the most frequently described delivery format [21, 60, 62–64, 68, 70–73, 78] with four studies describing blended learning approaches [69, 74, 76, 79]. Tele-mentoring [61] and simulation activities [67, 75, 77] were among more contemporary approaches whereas two of the oldest studies described CD-ROM training [65, 66]. Case-based instruction was the most frequently described instructional strategy [61, 62, 64, 65, 67, 69, 70, 73–76, 79]. Video-modelling [21, 68, 77] and reflective activities [65, 74, 79] were also described. Video was a popular mode of information delivery [21, 60, 66, 68, 70–74, 76–79]. Real-life videos including people with dementia, HSCPs and dementia experts were common video attributes [60, 71, 73]. Modes of information delivery were not always specified within the primary studies; however, graphics, text or audio were common multimedia choices [21, 64–68, 70–78]. Where online discussions were described [61, 64, 74, 76, 79]; three studies specified the use of asynchronous discussion boards [64, 74, 76] and one described a synchronous chat facility [76]. Assessment of learning frequently included assessments, quizzes or multiple choice questions with immediate feedback being an obvious benefit of TEDE. Hyperlinked text was occasionally included for access to external resources. Printed material was not a common resource characteristic.

#### Educational content

The educational content varied across healthcare settings. Table 6 consolidates the main learning outcomes where described in primary studies.

**Table 6:** Educational content

Setting	Educational content	Sources
Primary care	• Recognising dementia • Diagnosing dementia • Prescribing medication • Dementia progression • Care management	Bentley, Kerr et al. [60] Luconi [64] Vollmar, Mayer et al. [69]
Long-term care	• Introduction to dementia • Activities of daily living • Communication • Reacting skills • Redirection skills • Using reminiscence • Understanding dementia-related behaviours • Behaviour management • Pain management • Coping with challenging situations	Chao, Kaas et al. [79] Hobday, Savik, and Gaugler [71] Hobday et al. [72] Irvine, A. B., Bourgeois et al. [68] Irvine, A. Blair, Beaty et al. [21] Jones, Moyle [62] Tsai, Kitch et al. [77]
Hospital staff	• Introduction to dementia • Understanding dementia-related behaviours • Communication • Wandering and falls	Hobday, Gaugler & Mittelman [73]
Nursing students	• Psychosocial, cultural, cognitive, and spiritual development • Person-centred care • Understanding cognitive and functional change • Cognitive assessment • Communication • Activities of daily living and maximising independence • Understanding dementia-related behaviours • Distress behaviours • Dementia progression • Critical thinking skills • Involving family members and friends	Cobbett, Redmond et al. [74] Kimzey, Mastel-Smith et al. [63] Rababa, Masha'al [67] Ruiz, Smith et al. [66]
Medical students	• Understanding different types of dementia • Pathophysiology • Clinical presentation • Dementia diagnosis • Differential diagnosis • Treatment	Helms, Denson et al. [78] Matsumura, Shinno et al. [75] Westmoreland, Counsell et al. [70]

### Qualitative findings

There were variations in qualitative research objectives and context with prominent difference between pre-TEDE perceptions of dementia care and education [60, 63] and post-TEDE perceptions [60–62]. This resulted in two distinct groups which were synthesised independently. A structured summary of the qualitative evidence is presented with the initial codes and resulting themes generated in the synthesis (Additional file 6).

#### Pre-TEDE perceptions on dementia education and training

##### Theme 1. Existing strengths and experience

The qualitative data revealed that learners often bring existing knowledge and personal experience to their dementia education. One student stated that she ‘... *cares about learning more because family member has disease*’ [63]*.* Existing dementia knowledge was also influenced by media sources with informal learning accounting for some basic proficiency and caregiving competencies among less experienced practitioners [63]. Experiential variation also resulted from differences in professional exposure [60].

##### Theme 2. Knowledge gaps and uncertainty

Nursing students who were anxious about providing care to people with dementia were keen for more knowledge before engaging in practice. Students often held negative perceptions about dementia and perceived experiential learning to be necessary, as one student suggested: ‘*it would be helpful to interact and gain experience with people with AD*’ [63]*.* Another student requested ‘*more knowledge before experiencing people with AD so they [sic] would know what to expect*’ [63]. Practice-based experiences were not sufficient for primary care practitioners who managed their uncertainty by referring people with dementia to specialists for further investigation and diagnosis [60].

#### Post-TEDE perceptions on dementia education and training

##### Theme 1. Developing core competence and expertise

Practitioners frequently reported new knowledge and skills following TEDE. Learner gains also included positive attitudinal change and the application of new behaviours in practice [60–62]. Practitioners developed dementia awareness, confidence and increased self-efficacy to perform new skills, as one primary care physician highlighted: ‘*What I found very helpful was knowing that you can confidently assess a patient in general practice for dementia and actually start treatment in general practice and looking at the patient holistically*’ [60]*.* Learning gains were associated with developing dementia expertise to a level to which it could then be shared with others [60, 61].

##### Theme 2. Involving relevant others in TEDE

Participant perceptions following a tele-mentoring programme with significant input from dementia professionals provided valuable evidence for TEDE instructional methods. One learner commented: ‘*I liked having access to people with specialist knowledge and experience that was very helpful*’ [61]*.*

Participants enjoyed learning in online groups, which was considered important to reduce feelings of professional isolation and for maintaining motivation. Practitioners also enjoyed multidisciplinary perspectives which provided reassurances on their existing practice. A sense of community was considered to be a key benefit. Group work was not universally accepted with reticence noted among some practitioners who feared exposing perceived challenges to a diverse audience [61]. Isolation was also perceived to be problematic where interactivity was not possible [62].

##### Theme 3. Optimising feasibility

Forward planning was considered important for successful TEDE. Planning for staff cover and time away from clinical responsibilities were key considerations, as were advance preparations for learning activities. Protected learning time was advocated to avoid interruptions during TEDE; however, capacity for protected learning was considered to be dependent on the healthcare setting and clinical demands. Convenience and flexibility were considered to be benefits of TEDE which eliminated the need for travel, expenses, and time away from clinical practice as one participant confirmed: ‘*the convenience of, you know, being able to … dial in from … my laptop in work is very helpful … for the two of us contributing here today up in [Trust]*, *having to get down on a weekly basis to something in Belfast you know is not … feasible*’ [61]*.* Technical problems were perceived to be an occasional barrier to TEDE.

### Quantitative and qualitative evidence

Evidence from quantitative and qualitative data sources provided support for TEDE as an effective approach for knowledge and skills development among HSCPs. There was consensus across the research paradigms that TEDE can also support attitudinal and behavioural change. The quantitative evidence was weaker at higher levels of Kirkpatrick’s framework due to smaller samples and a relatively higher incidence of non-significant effects. Similarly, results due to training were seldom described in the qualitative evidence, although learning and experiential developments were highlighted which may have indirect influence on broader outcomes. The qualitative data highlighted that practitioners are likely to bring some existing dementia knowledge and skills and illustrated confounding factors including demographic variation and media influence that were not considered in the primary quantitative research. The relative profusion of quantitative evidence provided more support for TEDE within multiple practice contexts.

The inductive approach to qualitative synthesis established additional constructs that were not accessible from the quantitative data using Kirkpatrick’s model. In general, the qualitative evidence provided a broader competency and proficiency context and highlighted additional concepts such as *awareness* and *confidence* that did not easily fit into the pre-defined quantitative framework. Self-efficacy was established as a unique and important construct which positions it as a crucial factor for student motivation and improved behaviours in practice [83]. Practitioner expertise following TEDE emerged as a key theme; however, TEDE may only provide foundations for the development of expertise which is likely to require additional experience in practice [84]. The need for experiential learning was highlighted by novice practitioners in particular in order to address fear and uncertainties towards dementia care. This was supported by quantitative evidence where experiential learning was associated with significantly improved learning outcomes compared to TEDE [63].

Primary quantitative studies frequently detailed instructional design strategies and information delivery methods. However, the effects from specific pedagogy were not evaluated; therefore, TEDE effectiveness was attributed to the overarching delivery method. Several studies acknowledged this limitation and incorporated survey reports for more specific feedback of learner preferences. The qualitative evidence provided more focus on instructional design methods and highlighted group learning and access to dementia experts as key pedagogical strategies. Learning with and involving relevant others in TEDE emerged as a key theme which was contextually related to a highly interactive tele-mentoring programme where group work was key.

The qualitative evidence provided additional insights into TEDE through the identification of barriers and facilitators. Convenience and flexibility emerged as the key facilitating factors which are widely reported in the educational technology literature [25, 32, 85, 86]. The potential for technical issues and need for organisational support were potential barriers.

## Discussion

This review aimed to establish the characteristics of effective TEDE for HSCPs. The findings were based on 21 studies which provided support for TEDE to improve dementia knowledge, skills, and care attitudes among HSCPs from multiple practice contexts. Confidence in the findings was undermined by limitations in the methodological quality of quantitative studies. RCTs incurred negative quality judgments due to incomplete outcome data and issues with intervention non-adherence. Randomisation processes were generally well described which mitigates allocation bias and supports a causal inference between TEDE and the learning outcomes. However, unlike clinical trials, where the variables of interest can be tightly controlled, randomisation may not adequately control for other sources of error that are common in educational research. Frequent ‘confounders’ include changes in participant motivation, effects of other (non-intervention) training experiences and contextual factors [87]. Confounding also had a substantial negative impact on quality judgments in quantitative non-randomised studies. These extraneous variables appear to be pervasive in educational research which may explain why several TEDE studies described additional evaluation strategies that transcend cause and effect by incorporating context and experience through the integration of programme evaluations and mixed research methods. Crucially, the omission of confounding, as a quality indicator, would have increased the overall quality of the evidence from low to moderate. This brings in to question the relevance of generic appraisal tools (e.g. MMAT) to the educational research context. Confounding bias was not the only concern. MMAT appraisal resulted in frequent ‘Can’t tell’ judgments for participant representativeness despite several evaluations describing *intentionally* pragmatic sampling methods involving accessible learner cohorts. In contrast, issues from outcome measurement may have greater relevance to the educational research context as outcome measures need to accurately reflect instructional content. Specific tools for quality assessment of educational research studies have been developed. For instance, the Medical Education Research Study Quality Instrument (MERSQI) is a valid and reliable tool designed to measure the quality of experimental, quasi-experimental, and observational studies [88]. MERSQI assigns higher values to RCTs—but it does not include control of confounders as a specific quality indicator. It puts emphasis on the number of institutions studied and response rates, type of data, validity of evaluation instruments, data analysis techniques and key educational outcomes—which are well-aligned with Kirkpatrick’s model. The qualitative evidence was considered to be of higher methodological quality overall. Judgements on the quality of the qualitative studies were based on broad quality domains that are likely to have relevance to multiple research contexts.

Online learning was the most frequently described delivery method in TEDE. Case-based instruction was frequently used within training programmes based on real, virtual or text-based patient cases—which can support the application of theory into practice [89]. E-simulations were contemporary case-based innovations enabling learners to rehearse dementia care skills in a safe environment before application in practice [90]. Future research might explore innovative approaches to develop practitioners’ affective empathy in case-based learning. For instance, positive dementia care attitudes can often result from an appreciation of personhood [91]. Ethnodramas present real-life video cases that promote person-centred care through emotional engagement in the lives of people with dementia. This approach has been used effectively with large groups of healthcare staff and resulted in positive organisational culture change [92]. Incorporating narratives from the arts and humanities (such as film) may also develop practitioners’ awareness, providing insights into ‘what it is like’ for the person experiencing the condition [93]. Distinct from instructional methods, videos, graphics, text and audio were common TEDE multimedia. These media have specific attributes that may enhance learning processes [94]. Educators are encouraged to provide clear justifications for multimedia choices, in relation to how the human mind works and how they can be applied to optimise dementia education [95].

The literature confirmed differences in learning outcomes between practitioner groups which supports recommendations for role relevant dementia education [35]. It is however important that generic core competencies such as person-centred care and communication skills are not overlooked. Experiential variations based on professional, educational, and personal exposure to dementia are all likely to influence more specialised training needs. Future research might consider how technology can be harnessed to respond to individual learning requirements. Adaptive learning technology is an approach which allows users to enter personal data and make choices which alters pathways within programmes to produce material that is relevant [96]. This type of approach was described in a TEDE intervention for informal caregivers, volunteers and professional caregivers [97]. Educators, researchers and instructional designers might explore options for more learner-centred TEDE that is sensitive to existing experience. Practitioners must also have appropriate digital experience and skills to engage with TEDE. Despite requirements for the development and sustainment of digital competency from professional regulators [98], there are significant degrees of anxiety, and even resistance, from HSCPs around the professional use of digital technologies [99]. It is essential that all learners can engage equitably in TEDE and healthcare organisations must recognise digital literacy as a core skill for HSCPs and assist in the development of digital competence [100]. Interventions that are simply easy to use are unlikely to be sufficiently equitable for practitioners with established digital skills who may thrive using more advanced systems.

Peer discussions provide a critical dimension to learning processes regardless of whether they are online or in the traditional classroom [101]. Online learning communities can foster a sense of social connection and are valuable spaces for productive dialogue as learners often prefer online discussions to face-to-face conversations [25]. Learning communities in TEDE were well received by practitioners and may provide opportunities for professional and peer support, debrief and reflection. Reflection was a frequently described pedagogical strategy which can develop depth of understanding and have a wider impact on learning than simply acquiring new knowledge and skills [102]. It has particular relevance to dementia education given the emotional and psychological implications of practice and may play an important role in cultivating practitioners’ affective empathy where conventional ‘testing’ models are not appropriate. Future research might aim to understand the skills and levels of professional moderation required to support reflection and debrief in online TEDE communities. The strengths and limitations of synchronous and asynchronous discussion forums would also merit further investigation. Communication and collaboration are key benefits from Web 2.0; however, it is not clear if social networking has a role in augmenting learning conversations. More research would help determine the suitability of social media in TEDE including the potential for confidentiality issues and professionalism concerns [103].

Future TEDE research might aim to understand how key attributes—convenience and flexibility—can support practitioners who experience barriers to professional development as a result of access limitations. Rural practitioners may gain particular benefit; however, barriers to TEDE in rural areas would also require consideration given the established urban-rural digital divide [104]. TEDE research, like all educational research, is highly context dependent and the ‘real world’ influence cannot be easily eliminated [105]. This suggests that future research might shift focus from effectiveness and generalisable solutions towards a more nuanced understanding of TEDE and the complex environments in which it is situated [106].

### Limitations

This review has several limitations. Firstly, the identification of an additional study in citation searching implied limitations to the search strategy. A possible cause for this was the use of the unexploded thesaurus term ‘education’ and the omission of synonymous terms including keywords and relevant variations. The rationale for the search strategy was described at the protocol stage and aimed to increase the precision of the search by reducing irrelevant results from the diverse array of educational subheadings that exist within educational subject headings. To compensate for this, the search strategy was formulated to include additional database-specific thesaurus terms relating to ‘technology-enabled learning’. For instance, ‘Computer-Assisted Instruction’ and ‘Education, Distance’ were thesaurus terms identified in MEDLINE, whereas the educational resource ERIC offered additional and alternative terms (i.e. ‘Blended Learning’, ‘Electronic Learning’, ‘Distance Education’, ‘Multimedia Instruction’, ‘Web Based Instruction’, ‘Online Courses’ and ‘Computer Assisted Instruction’). The associated keywords were developed to contain both a technological and educational component (e.g. online *and* education) which were formulated with appropriate variations and translated for functionality across the multiple databases. However, the technological components identified may not have been inclusive and variations on the educational component were not consistently integrated. The development of the search strategy was an iterative process guided by a subject librarian and the final search terms were modifications based on multiple search efforts and related retrieval information. It is worthwhile to note that there can be diminishing returns for ongoing search efforts, i.e. after a certain stage, each additional unit of time invested in searching returns fewer references that are relevant to the review [56]. It was however important to the rigorous conduct of this systematic review that the final search strategy was presented, and limitations acknowledged, for increased clarity, transparency and future reproducibility.

The review process included strategies to optimise the identification of relevant studies. Searching multiple bibliographic databases and the reference lists of eligible studies helped to achieve reliable accounts of TEDE characteristics and effectiveness. Bias may have been further minimised through the integration of additional supplementary search methods, e.g. contacting study authors for details of other potentially relevant studies. Decisions to perform additional search methods were influenced by time and resources available; therefore, study authors were not contacted as this can be time consuming with low response rates and no guarantee of obtaining relevant information [107]. It is nonetheless important to highlight this as a limitation and potential source of bias.

The study selection process was comprehensive; however, two papers entitled ‘resources’ could not be located and were discarded at the screening stage. Title and abstract screening, data extraction and quality assessment were mostly conducted by one reviewer due to resource limitations. Risks of selection bias were mitigated by second reviewers screening a percentage of titles and abstracts. Second reviewers also appraised a proportion of studies to mitigate error and subjective judgment in quality assessment. Language bias could also have been introduced in the review as it was exclusively based on English-language reports. Furthermore, studies published in a foreign language were simply discarded at the screening stage which did not permit insights into the extent and effects of this potential bias.

Methods to assess the overall quality of included studies were adapted from guidance that suggests review authors identify the most important domains (‘key domains’) that feed into assessments [56, 57]. In the current review, all domains were considered to be important as issues with confounding bias became apparent *following* quality assessment. It is worth reiterating that the exclusion of confounding bias, as an unimportant domain, would have resulted in greater confidence in the quality of the evidence overall.

The effectiveness of TEDE was established from vote counting techniques as heterogeneity precluded meta-analysis. This did not include information on the magnitude of effects or differences based on the relative size of studies [82]. Quality judgments were not factored into the analysis of effectiveness; therefore, outcome effects require cautious interpretation. Experimental studies were appropriate to establish intervention effects; however, additional TEDE characteristics may have been identified from descriptive research. This review did not therefore provide a comprehensive account of innovative TEDE practices. It is also important to highlight that effectiveness was attributed to overarching delivery methods and the effects of specific pedagogy were not established. The pedagogical characteristics identified are unlikely to be fully representative due to underreporting in primary studies. Further, broad conclusions on overall TEDE effects were reported despite multiple intervention types being described.

The complexities of TEDE and educational research represented challenges for strict fidelity to the review protocol. It was not possible to report on the full range of secondary outcomes due to reporting limitations in primary studies. Qualitative evidence was intended to complement quantitative evidence; however, the dependency on robust qualitative evidence resulted in a more complete and contextual portrayal of TEDE and shift from complementarity towards triangulation—which omitted several accounts of user satisfaction and subjective experience with TEDE [21, 64, 66–68, 71–75, 77]. Analytic themes were not generated from the qualitative synthesis as descriptive themes sufficiently represented the qualitative data, providing support for TEDE and future research. The Grading of Recommendations Assessment, Development and Evaluation (GRADE) approach to comment on the certainty of the quantitative findings was not included due to highly heterogenous study data that were not amenable to the synthesis techniques required for robust appraisal. The GRADE-CERQual approach was not applied to qualitative findings as MMAT appraisal provided sufficient confidence in the quality of the limited qualitative evidence.

## Conclusions

TEDE is a convenient and flexible teaching and learning approach that can develop dementia care competence and confidence among various HSCPs. Translation of theory into practice is optimised by case-based instruction using various multimedia that can emulate real-life situations providing a useful proxy for traditional and experiential learning. Reflective activities and debrief are achievable following simulated or instructive activities; however, learning networks for group discussions may require moderation and optimal communication platforms need to be established. Equitable engagement will be critical to the future success of TEDE which may require protected learning time, technical support and sustainment and development of digital skills among practitioners. Future TEDE research might acknowledge critical differences between clinical and educational research and place greater emphasis on specific pedagogy within interventions and the role of TEDE in ameliorating organisational and environmental limitations including barriers to traditional dementia education.

## Supplementary Information

**Additional file 1.**

**Additional file 2.**

**Additional file 3.**

**Additional file 4.**

**Additional file 5.**

**Additional file 6.**

## Data Availability

Not applicable.

## Abbreviations

AD: Alzheimer’s disease
GRADE: Grading of Recommendations Assessment, Development and Evaluation
GRADE-CERQual: Confidence in the Evidence from Reviews of Qualitative research
HSCP: Health and social care practitioner
MERSQI: Medical Education Research Study Quality Instrument
MMAT: Mixed Methods Appraisal Tool
PRISMA: Preferred Reporting Items for Systematic Review and Meta-Analysis
PROSPERO: International Prospective Register of Systematic Reviews
RCT: Randomised controlled trial
TEDE: Technology-enabled dementia education
