# Supplementary material for: The characteristics of effective technology-enabled dementia education: a systematic review and mixed research synthesis
Source: Syst Rev. 2022 Feb 23;11:34. doi: 10.1186/s13643-021-01866-4 (PMC8865181; doi:10.1186/s13643-021-01866-4)
Supplement: Supplementary file 3 — Additional file 3. [file 13643_2021_1866_MOESM3_ESM.docx]

**Additional file 3. Sample Data Extraction Forms**

**Quantitative Studies**

**Author & Title:**

| *[Insert additional comments]*   \| MMAT *[Insert Study Design Category]* \| Authors' judgement \| Support for judgement \| \| --- \| --- \| --- \| \| *Insert relevant methodological quality criteria* \|  \|  \| \| *Insert relevant methodological quality criteria* \|  \|  \| \| *Insert relevant methodological quality criteria* \|  \|  \| \| *Insert relevant methodological quality criteria* \|  \|  \| \| *Insert relevant methodological quality criteria* \|  \|  \|   *[Insert additional comments]* | |
| --- | --- | --- | --- | --- | --- | --- | --- | --- | --- | --- | --- | --- | --- | --- | --- | --- | --- | --- | --- |
| Citation |  |
| Study Design |  |
| Aims |  |
| Country |  |
| Ethics |  |
| Participant Characteristics |  |
| Setting |  |
| Participant Demographics |  |
| Results of Quality Assessment |  |
| Sampling Approach |  |
| Inclusion Criteria |  |
| Exclusion Criteria |  |
| Data Collection Instruments |  |
| Internal Validity/ Reliability |  |
| Data Analysis Methods |  |
| Power Calculation |  |
| Response Rate / Outcome Data |  |
| Intervention |  |
| Educational Content |  |
| Technical Characteristics |  |
| Pedagogical Approach |  |
| Duration |  |
| Educational/ Pedagogical Theories |  |
| Comparator/ Control Group |  |
| Learner Satisfaction |  |
| Knowledge |  |
| Skills |  |
| Attitudes |  |
| Behaviours |  |
| Results |  |
| Educator experience |  |
| Functionality |  |
| Technical Support |  |
| Usability (PU/ PEU) |  |
| Cost-effectiveness |  |
| Attrition |  |
| Opportunity for reflection |  |
| Curricular Basis (Dementia) |  |

**Qualitative Studies**

**Author & Title:**

| \| MMA1. Qualitative Studies \| Authors' judgement \| Support for judgement \| \| --- \| --- \| --- \| \| Is the qualitative approach appropriate to answer the research question? \|  \|  \| \| Are the qualitative data collection methods adequate to address the research question? \|  \|  \| \| Are the findings adequately derived from the data? \|  \|  \| \| Is the interpretation of results sufficiently substantiated by data? \|  \|  \| \| Is there coherence between qualitative data sources, collection, analysis, and interpretation? \|  \|  \| | |
| --- | --- | --- | --- | --- | --- | --- | --- | --- | --- | --- | --- | --- | --- | --- | --- | --- | --- | --- | --- |
| Citation |  |
| Aims / Research question |  |
| Country |  |
| Ethics |  |
| Participant Characteristics |  |
| Intervention |  |
| Setting |  |
| Results of Quality Assessment |  |
| Sampling Approach |  |
| Sample (numbers) |  |
| Qualitative approach / Epistemology |  |
| Data Collection Methods |  |
| Data Analysis Methods |  |
| Educational Content |  |
| Technical Characteristics |  |
| Pedagogical Approach |  |
| Educational/ Pedagogical Theories |  |
| Qualitative Themes |  |
| Qualitative Data | *[Insert all qualitative data from ‘findings’ or ‘results’ sections]* |
