# Supplementary material for: The characteristics of effective technology-enabled dementia education: a systematic review and mixed research synthesis
Source: Syst Rev. 2022 Feb 23;11:34. doi: 10.1186/s13643-021-01866-4 (PMC8865181; doi:10.1186/s13643-021-01866-4)
Supplement: Supplementary file 4 — Additional file 4. [file 13643_2021_1866_MOESM4_ESM.docx]

**Additional file 4. Characteristics of Included Studies**

**Bentley, Kerr et al. 2019 (60)**

| Methods | Single groups pre- and post-tests | | |
| --- | --- | --- | --- |
| Participants | International medical graduates (IMG) and practice nurses (PN) | | |
| Setting | Primary care | | |
| Country | Australia | | |
| Intervention | Recognising, diagnosing and managing dementia in general practice. Interactive, online educational resource. | | |
| Comparator | Not applicable | | |
| Outcomes | Knowledge  Attitudes | | |
| Educational Content | - Recognising dementia in general practice - Diagnosing dementia in general practice - How does dementia progress - Managing dementia in general practice | | |
| Pedagogical Approach | Online learning using video, assessment questions, and additional learning sources.   - Videos have a conversational format with discussions between GPs, nurses, carers, and people living with dementia. The discussions focus on challenges facing primary care professionals in general practice. Other dementia clinicians and experts join the conversation to share current practice and research and to emphasise key information | | |
| Technological Specifications | Online educational resource - not otherwise specified. | | |
| Educational Theory | None specified. | | |
|  | | | |
| **Quality Criteria (MMAT 3)** | | **Author Judgement** | **Support for Judgement** |
| Are the participants representative of the target population? | | Can’t tell | The sampling approach did not ensure representativeness. |
| Are measurements appropriate regarding both the outcome and intervention (or exposure)? | | Can’t tell | Validity of DKAS and GPACS-D were reported from previous studies. |
| Are there complete outcome data? | | Yes | Incomplete data for 18.5% of participants who completed online modules was satisfactory based on arbitrary threshold (20%). |
| Are the confounders accounted for in the design and analysis? | | No | Confounders were not accounted for with significant time difference between pre- and post-tests. |
| During the study period, is the intervention administered (or exposure occurred) as intended? | | Can’t tell | There was a lack of assurances re intervention controls including the place of intervention completion. |
| **Quality Criteria (MMAT 1)** | | **Author Judgement** | **Support for Judgement** |
| Is the qualitative approach appropriate to answer the research question? | | Yes | Semi-structured interviews were thematically analysed. |
| Are the qualitative data collection methods adequate to address the research question? | | Can’t tell | The form of the data was not described. |
| Are the findings adequately derived from the data? | | Can’t tell | Coding methods were not specified. |
| Is the interpretation of results sufficiently substantiated by data? | | Can’t tell | Themes / quotations were not reported in pre interview data and themes were not clear in post interviews. |
| Is there coherence between qualitative data sources, collection, analysis and interpretation? | | Yes | There was no evidence of non-coherence between data sources, collection, analysis and interpretation. |

**Chao, Kaas et al. 2016 (79)**

| Methods | One Group Repeated Measure | | |
| --- | --- | --- | --- |
| Participants | Registered Nurses or Licensed Practice Nurses | | |
| Setting | Long term care | | |
| Country | Taiwan | | |
| Intervention | Advanced innovative internet-based communication education  program: Promoting communication between nurses and patients with dementia | | |
| Comparator | Not applicable | | |
| Outcomes | Knowledge  Attitudes  Skills  Behaviours  Results | | |
| Educational Content | The core competencies of the AIICE program included:   - knowing the behaviour patterns of patients - using reminiscence - using scenario internet-based self-directed learning - executing real-world practice and experience sharing - Module 1: Classroom lecture (behavioural and psychological symptoms of dementia and communication strategies) - Module 2: Workshop on reminiscence - Module 3. Internet-based (scenario-based self-learning using video clips showing negative approaches to communication) with questions and online peer discussion - Module 4: Reflective journaling of experiences of communicating with patients with dementia in practice using (internet-based) 360-degree feedback system including reflective discussions with peers and program instructor | | |
| Pedagogical Approach | Blended learning approach using classroom / workshop activity in combination with internet-based learning using videos, quizzes, online discussion and 360-degree reflective feedback. | | |
| Technological Specifications | Internet-based – not otherwise specified. | | |
| Educational Theory | Adult learning theory. | | |
|  | | | |
| **Quality Criteria (MMAT 3)** | | **Author Judgement** | **Support for Judgement** |
| Are the participants representative of the target population? | | Can’t tell | Convenience sample. |
| Are measurements appropriate regarding both the outcome and intervention (or exposure)? | | Can’t tell | There was good evidence of psychometric evaluation for most outcome measures. Its was not clear if the RMBP-C has been content validated in context. |
| Are there complete outcome data? | | Yes | There was missing data for 2% of participants at 16 weeks which is satisfactory based on the arbitrary threshold (20%). |
| Are the confounders accounted for in the design and analysis? | | No | Confounders were not included in analysis with significant time difference and opportunity for maturation effects between baseline and final tests. |
| During the study period, is the intervention administered (or exposure occurred) as intended? | | Can’t tell | The authors reported that several participants failed to fully participate in follow up internet discussions and reflection assignments. |

**Cobbett, Redmond et al. 2016 (74)**

| Methods | Non-equivalent control group pre- and post-test | | |
| --- | --- | --- | --- |
| Participants | Nursing students | | |
| Setting | University campus | | |
| Country | Canada | | |
| Intervention | Alzheimer’s disease and other associated dementias care course (ADODCC): Adapted online course | | |
| Comparator | Non-participation in ADODCC | | |
| Outcomes | (Knowledge and skills) | | |
| Educational Content | ADODCC is part of a healthy aging nursing course which examines psychosocial, cultural, cognitive, and spiritual development of adults, health promotion and implications for nursing practice. | | |
| Pedagogical Approach | Online course with modules that include readings and online specific activities such as discussions, wikis, personal journals, and quizzes.  The first eight modules were delivered online and the final module was delivered through a face-to-face presentation.  A certificate is available on completion. | | |
| Technological Specifications | Online format – not otherwise specified. | | |
| Educational Theory | None specified. | | |
|  | | | |
| **Quality Criteria (MMAT 3)** | | **Author Judgement** | **Support for Judgement** |
| Are the participants representative of the target population? | | Can’t tell | Convenience sampling where completion of pre- and post tests was discretionary. |
| Are measurements appropriate regarding both the outcome and intervention (or exposure)? | | Can’t tell | Two subscales (comprehension and critical thinking) were below acceptable alpha levels and may not be reliable. |
| Are there complete outcome data? | | Can’t tell | It was not clear if all participants who completed pre-tests completed post-tests. |
| Are the confounders accounted for in the design and analysis? | | No | Potential confounders were identified with relevance between groups. Intervention duration significant with potential for additional confounders (maturation effects) between pre- and post tests. |
| During the study period, is the intervention administered (or exposure occurred) as intended? | | Yes | There was no evidence that the intervention was not administered as intended. |

**De Witt Jansen, Brazil et al. 2018 (61)**

| Methods | Single groups pre- and post-test | | |
| --- | --- | --- | --- |
| Participants | Physicians, nurses, and health care assistants | | |
| Setting | Primary, secondary, nursing home, and hospice | | |
| Country | UK | | |
| Intervention | Tele mentoring to enhance assessment and management of pain in advanced dementia (based on Project ECHO model) | | |
| Comparator | Not applicable | | |
| Outcomes | (Knowledge and Skills) | | |
| Educational Content | - Managing challenges of routes of administration in pain management for people with advanced dementia - Non-pharmacological aspects of pain management in advanced dementia - Pain assessment in advanced dementia - Pharmacology in advanced dementia - Differentiating the behavioural indicators of pain from anxiety, agitation and other non-pain related behaviours in dementia | | |
| Pedagogical Approach | Tele mentoring approach involving clinicians with relevant patient experience and clinical knowledge providing brief, focused didactic training on the clinical area. Learners (typically one or two) present anonymised real patient cases for discussion. Discussions provide opportunity for shared decision-making between specialists and learners. At the close of discussion, facilitators summarise a proposed treatment plan/ guidance. | | |
| Technological Specifications | Clinics were digitally recorded using video with audio. Participants attending at the hub or virtually (place of work) using Zoom videoconferencing technology. | | |
| Educational Theory | Community of Practice Theory emphasises the importance of learning through continuous participation in a collaborative community consisting of peer learners and expert individuals. | | |
|  | | | |
| **Quality Criteria (MMAT 3)** | | **Author Judgement** | **Support for Judgement** |
| Are the participants representative of the target population? | | Yes | The study evaluated the impact of Project ECHO on participants using a census sampling approach. |
| Are measurements appropriate regarding both the outcome and intervention (or exposure)? | | Can’t tell | There was evidence of content validation of the outcome measures. Internal consistency was not described. |
| Are there complete outcome data? | | Can’t tell | Disparities between amounts of participating HCPs (table 3) and outcome data were not clear. |
| Are the confounders accounted for in the design and analysis? | | No | Confounders were not accounted for. |
| During the study period, is the intervention administered (or exposure occurred) as intended? | | Can’t tell | There authors reported technical issues and insufficient time for case familiarity before clinics. |
| **Quality Criteria (MMAT 1)** | | **Author Judgement** | **Support for Judgement** |
| Is the qualitative approach appropriate to answer the research question? | | Yes | Focus groups and thematic analysis. |
| Are the qualitative data collection methods adequate to address the research question? | | Yes | Focus groups were video-recorded, and audio data transcribed verbatim, checked and verified for accuracy. |
| Are the findings adequately derived from the data? | | Yes | The data were analysed using Braun and Clarke’s model of thematic analysis. |
| Is the interpretation of results sufficiently substantiated by data? | | Yes | Themes were presented and quotations justified themes. |
| Is there coherence between qualitative data sources, collection, analysis and interpretation? | | Yes | There was no evidence of non-coherence between data sources, collection, analysis and interpretation. |

**Downs, Turner et al. 2006 (65)**

| Methods | Cluster randomised controlled before and after study | | |
| --- | --- | --- | --- |
| Participants | GP practices | | |
| Setting | Primary Care | | |
| Country | UK | | |
| Intervention | - Educational tutorial on CD-ROM - Decision support software - Practice based workshops | | |
| Comparator | No intervention (data collection only) | | |
| Outcomes | Behaviours | | |
| Educational Content | The authors reported in 2006 that the curriculum for the electronic tutorial was available at Alzheimer’s society website – not otherwise specified. | | |
| Pedagogical Approach | The electronic tutorial allowed for a mode of learning from case analysis, with the emphasis on reflecting on knowledge and revisiting particularly difficult and complex clinical problems. The tutorial was an “electronic book,” with an indexing system that allows easy access to different themes, and hypertext links that allowed the reader to move easily from one subject to another. | | |
| Technological Specifications | Educational tutorial on CD-ROM – not otherwise specified. | | |
| Educational Theory | The educational interventions reflected different approaches to adult learning. | | |
|  | | | |
| **Quality Criteria (MMAT 2)** | | **Author Judgement** | **Support for Judgement** |
| Is randomization appropriately performed? | | Yes | An individual outside the research team used a computer-generated program to randomise participating practices to receive one of the three interventions or to act as control. The research team and practices remained blinded to randomisation until after baseline data had been collected. |
| Are the groups comparable at baseline? | | No | While profiles of age and sex were similar across all four arms, the proportion of patients in residential care was lower in the decision support software and control arm practices. |
| Are there complete outcome data? | | No | There was missing (patient record) data (34%) which is unacceptable based on the arbitrary threshold (20%). |
| Are outcome assessors blinded to the intervention provided? | | No | This was an unblinded cluster randomised controlled study. |
| Did the participants adhere to the assigned intervention? | | Yes | There was no evidence of non-adherence to the interventions / not applicible. |

**Helms, Denson et al. 2009 (78)**

| Methods | Nonrandomised with control group | | |
| --- | --- | --- | --- |
| Participants | Medical students | | |
| Setting | Higher Education | | |
| Country | USA | | |
| Intervention | E-module: Neurology and Dementia: Psychological Aspects of Care  (adjunct to neurology clerkship materials) | | |
| Comparator | Neurology clerkship students not electing to participate in e-module | | |
| Outcomes | Skills | | |
| Educational Content | Not specified.   - The material was well integrated with the clerkship’s didactic learning related to the pathophysiology, clinical presentation, and medical therapies of dementia patients and, thus, was not disjointed from the curriculum | | |
| Pedagogical Approach | Hybrid learning approach including an e-module (intervention) in conjunction with didactic presentations of dementia as a clinical problem.   - E-modules included video segments hyperlinked to explanatory text and external resources, quiz, and module evaluation | | |
| Technological Specifications | E-module design incorporated multiple formats and media using the ANGEL e-learning management system as the electronic delivery platform. | | |
| Educational Theory | None specified. | | |
|  | | | |
| **Quality Criteria (MMAT 3)** | | **Author Judgement** | **Support for Judgement** |
| Are the participants representative of the target population? | | Can’t tell | Participation in geriatrics group was voluntary and sampling was not otherwise described. |
| Are measurements appropriate regarding both the outcome and intervention (or exposure)? | | Can’t tell | Validity / reliability was not reported. |
| Are there complete outcome data? | | Yes | Data was available for all neurology clerkship students and geriatric group volunteers. |
| Are the confounders accounted for in the design and analysis? | | No | Confounders were not described / accounted for. |
| During the study period, is the intervention administered (or exposure occurred) as intended? | | Can’t tell | There was lack of assurances re intervention controls including place / time of intervention completion. |

**Hobday, Savik, and Gaugler 2010 (71)**

| Methods | Single group pre- and post-test | | |
| --- | --- | --- | --- |
| Participants | Direct care workers | | |
| Setting | Nursing homes | | |
| Country | USA | | |
| Intervention | Internet-based multimedia education (IBME) dementia training resource | | |
| Comparator | Not applicable | | |
| Outcomes | Knowledge | | |
| Educational Content | - Introduction to dementia - Rethinking activities - Toileting | | |
| Pedagogical Approach | Internet based training using text, video, audio, and photographic content.   - Real life videos | | |
| Technological Specifications | Browser-based computer program using Adobe Flash. The program requires a Web browser (such as Internet Explorer or Netscape) and a Flash Player plug-in (downloadable as required). | | |
| Educational Theory | None specified. | | |
|  | | | |
| **Quality Criteria (MMAT 3)** | | **Author Judgement** | **Support for Judgement** |
| Are the participants representative of the target population? | | Can’t tell | The sampling approach did not ensure representativeness. |
| Are measurements appropriate regarding both the outcome and intervention (or exposure)? | | Can’t tell | There was limited evidence of content validation. The internal reliability of the knowledge test was ‘moderate’ (alpha = 0.61). |
| Are there complete outcome data? | | No | Outcome data was missing from 38% which is > arbitrary threshold of 20%. |
| Are the confounders accounted for in the design and analysis? | | No | Confounders were not accounted for in the analysis and there was a possibility of maturation effects between pre- and post-tests. |
| During the study period, is the intervention administered (or exposure occurred) as intended? | | Can’t tell | There was a lack of assurances re intervention controls as authors acknowledged that place / time of completion was not tracked. |

**Hobday et al. 2010 (72)**

| Methods | Single group pre- and post-test | | |
| --- | --- | --- | --- |
| Participants | Certified nurse assistants | | |
| Setting | Nursing homes and assisted living facility | | |
| Country | USA | | |
| Intervention | Internet-based, interactive, multimedia dementia educational program | | |
| Comparator | Not applicable | | |
| Outcomes | Knowledge | | |
| Educational Content | - Opener (necessary to guide users as they began using the prototype) - Introduction to Dementia (included information on the assessment and staging of dementia) - Introduction to behaviour management - Food and fluid Intake - Pain management in residents with dementia - Communicating with residents | | |
| Pedagogical Approach | Web-based learning using text, graphics, and video. | | |
| Technological Specifications | Web accessible via internet connection. | | |
| Educational Theory | None specified. | | |
|  | | | |
| **Quality Criteria (MMAT 3)** | | **Author Judgement** | **Support for Judgement** |
| Are the participants representative of the target population? | | Can’t tell | The sampling approach was not reported. |
| Are measurements appropriate regarding both the outcome and intervention (or exposure)? | | Yes | There was evidence of content validity and reliably of the knowledge test. |
| Are there complete outcome data? | | Can’t tell | It was not clear how many participants completed pre-tests. |
| Are the confounders accounted for in the design and analysis? | | No | Confounders were not accounted for and pre- to post-test time difference was unclear. |
| During the study period, is the intervention administered (or exposure occurred) as intended? | | Can’t tell | There was lack of assurances on intervention controls and authors reported that it was not clear where the training was completed. |

**Hobday, Gaugler & Mittelman 2017 (73)**

| Methods | Single group pre- and post-test | | |
| --- | --- | --- | --- |
| Participants | Nursing assistants (NA) and allied hospital workers (AHW) | | |
| Setting | Rural and metropolitan hospitals | | |
| Country | USA | | |
| Intervention | CARES Dementia-friendly Hospital (CDFH) online dementia training program | | |
| Comparator | Not applicable | | |
| Outcomes | Knowledge | | |
| Educational Content | - Introduction to dementia-friendly care - Communicating with patients - Dementia-related behaviour - Wandering and falls | | |
| Pedagogical Approach | Online learning using audio-narrated text, graphics, video interview, video scenarios, interactive text-entry, and case study scenarios asking learners to synthesize knowledge learned in real case scenarios.   - Audio-narrated text, in particular, is used to address NAs/AHWs at the appropriate literacy level. There is real, unscripted video footage of interactions between real patients with dementia, their families, and caregivers, as well as video interviews with real staff members and dementia experts | | |
| Technological Specifications | CDFH is delivered via an online website. CDFH was completed on NAs’ / AHWs’ own computers, tablets, or smartphones. | | |
| Educational Theory | - Research-based characteristics of effective adult learning mechanisms and principles were incorporated throughout modules including multiple examples to explain key points; material relevant to NAs’/AHWs’ work-related responsibilities; asking NAs/AHWs to engage with and actively use the information presented; and additional practice opportunities with note-taking devices, worksheets, and opportunities for reflective response - The development of CDFH was based on interactive design principles (ADDIE methodology: Analysis, Design, Development, Implementation, and Evaluation) | | |
|  | | | |
| **Quality Criteria (MMAT 3)** | | **Author Judgement** | **Support for Judgement** |
| Are the participants representative of the target population? | | Can’t tell | Convenience sample. |
| Are measurements appropriate regarding both the outcome and intervention (or exposure)? | | Can’t tell | Reliability was not reported. |
| Are there complete outcome data? | | Yes | The knowledge test outcome data appeared to be complete. |
| Are the confounders accounted for in the design and analysis? | | No | Confounders were not accounted for. Average pre- to post-test duration was 8 days (possible maturation effects). |
| During the study period, is the intervention administered (or exposure occurred) as intended? | | Can’t tell | CDFH was completed on participants own devices with insufficient information about intervention / testing controls. |

**Irvine, A. B., Bourgeois et al. 2007 (68)**

| Methods | Randomised controlled trial (with pre and post) tests | | |
| --- | --- | --- | --- |
| Participants | Nurse aides (NA) | | |
| Setting | Long term care | | |
| Country | USA | | |
| Intervention | Interactive multimedia program: Professional Dementia Care: Managing Aggression | | |
| Comparator | Control group who did not participate in training program | | |
| Outcomes | Knowledge  Attitudes  Skills | | |
| Educational Content | Content provided skills for approaching an agitated resident exhibiting potentially dangerous behaviours and for safely de-escalating the situation.  A.I.D approach:   - A = Assess - I = Investigate - D = Do something   The over-reaching philosophy of person-centred care was emphasized. | | |
| Pedagogical Approach | Internet training using storyboards incorporating graphics, video vignettes, and testimonials.   - After watching a video vignette (NA reaction to aggressive behaviour, the learner chose correct answer from a series of multiple-choice questions about the appropriateness of the NA’s response. Correct answers were reinforced, and incorrect answers were remediated with an explanation of why they were incorrect. The learner then saw correct modelling of how to deal with the aggressive situation. After viewing the testing vignette, users who responded incorrectly were re-tested on those vignettes until correct responses were elicited.   Text were written at 2^nd^-6^th^ grade reading level. | | |
| Technological Specifications | Internet training program – not otherwise specified. | | |
| Educational Theory | The authors report that research on adult learners suggests behaviour modelling training enhances learning and program effectiveness, which supported the instructional design in this intervention.  TRA and social cognitive theory applied for outcome measurement related to behavioural change. | | |
|  | | | |
| **Quality Criteria (MMAT 2)** | | **Author Judgement** | **Support for Judgement** |
| Is randomization appropriately performed? | | Can’t tell | Method of randomisation / allocation concealment were not specified. |
| Are the groups comparable at baseline? | | Yes | The treatment and control did not differ significantly on pre-test demographic variables. |
| Are there complete outcome data? | | Yes | Data was available for 62 participants completing both T1 and T2 assessments. |
| Are outcome assessors blinded to the intervention provided? | | Yes | Computerised outcome assessment. |
| Did the participants adhere to the assigned intervention? | | Can’t tell | Participation was unsupervised. |

**Irvine, A. Blair, Beaty et al. 2013 (21)**

| Methods | Within-subjects pre- and post-tests | | |
| --- | --- | --- | --- |
| Participants | Non direct care workers  (Includes nurses/LPNs) | | |
| Setting | Long term care | | |
| Country | USA | | |
| Intervention | Internet dementia-training program | | |
| Comparator | Not applicable | | |
| Outcomes | Knowledge  Attitudes  Skills | | |
| Educational Content | - Speaking Skills. Covered appropriate ways to greet and talk to a resident with dementia (e.g., say the resident’s name, and say your name; speak slowly; be patient) - Reacting Skills. Taught users to control their emotions if confronted by a resident (e.g., stay calm, acknowledge the resident’s emotions, don’t argue about reality, be patient) - Redirection Skills. Modelled ways to redirect residents to alternative activities (e.g., how to shift the topic based on what the resident said or what the caregiver knows about the resident) - Communication Cards. Showed how to print short personalized messages on note cards and how to present them to a resident for reassurance or redirection - When Bad Things Happen. Provided interactive education on how to deal with grief (i.e., due to death of a resident), stress, and verbal confrontations by a resident (i.e., racial, sexual, general harassment) | | |
| Pedagogical Approach | Internet training using video-modelling vignettes, right-way and wrong-way exemplars, testimonials, and narration.  Text written at a sixth-grade reading level. | | |
| Technological Specifications | Internet-based computer training – not otherwise specified. | | |
| Educational Theory | TRA and social cognitive theory applied for outcome measurement related to behavioural change. | | |
|  | | | |
| **Quality Criteria (MMAT 3)** | | **Author Judgement** | **Support for Judgement** |
| Are the participants representative of the target population? | | Yes | A total population sampling approach was used. |
| Are measurements appropriate regarding both the outcome and intervention (or exposure)? | | Can’t tell | There was variability in reliability reporting and no evidence of content validation. |
| Are there complete outcome data? | | Yes | There was missing data for 16% of total study participants at T_3_ which is acceptable based on an arbitrary threshold of 20%. |
| Are the confounders accounted for in the design and analysis? | | Yes | Some confounders were identified and T1 to T2 assessments assessed the plausibility of maturation and effects. |
| During the study period, is the intervention administered (or exposure occurred) as intended? | | Yes | There was no evidence that the intervention was not administered as intended. |

**Jones, Moyle 2016 (62)**

| Methods | Single group pre- and post-tests | | |
| --- | --- | --- | --- |
| Participants | Undergraduate nursing students; Registered nurses; Enrolled nurses; Personal care workers; Diversional therapists | | |
| Setting | University and residential aged care facilities | | |
| Country | Australia | | |
| Intervention | Online self-directed eLearning education intervention (based on the sexualities and dementia education resource for health professionals) | | |
| Comparator | Not applicable | | |
| Outcomes | Knowledge  Attitudes | | |
| Educational Content | - Intimacy, sexuality and sexual behaviour - Dementia and the expression of sexuality - Ethical considerations: policy guidelines development for sexualities and dementia in care settings - Developing sexualities and dementia policy guidelines for care practice | | |
| Pedagogical Approach | E-learning (online) using case studies, activities, and resources. | | |
| Technological Specifications | Online learning – not otherwise specified. | | |
| Educational Theory | None specified. | | |
|  | | | |
| **Quality Criteria (MMAT 3)** | | **Author Judgement** | **Support for Judgement** |
| Are the participants representative of the target population? | | Can’t tell | The sampling approach did not ensure representativeness and authors acknowledged sampling limitations. |
| Are measurements appropriate regarding both the outcome and intervention (or exposure)? | | Can’t tell | There was evidence of reliability for the ASKAS. SAID psychometric properties were undetermined. |
| Are there complete outcome data? | | Can’t tell | 42 participants completed the educational intervention. It was not clear if all participants completed pre- and post-tests. |
| Are the confounders accounted for in the design and analysis? | | No | There were attempts to identify some potential confounders – methods to control for confounders were not described. Additional confounders were possible from potential maturation effects (4 weeks between pre- and post-tests). |
| During the study period, is the intervention administered (or exposure occurred) as intended? | | Can’t tell | There was lack of assurances re intervention controls including place of intervention completion. |
| **Quality Criteria (MMAT 1)** | | **Author Judgement** | **Support for Judgement** |
| Is the qualitative approach appropriate to answer the research question? | | Yes | Individual semi-structured interviews with data analysis methods specified. |
| Are the qualitative data collection methods adequate to address the research question? | | Can’ tell | The form of the data was not described. |
| Are the findings adequately derived from the data? | | Can’ tell | Coding methods were not specified. |
| Is the interpretation of results sufficiently substantiated by data? | | Yes | There were quotes available to support all themes. |
| Is there coherence between qualitative data sources, collection, analysis and interpretation? | | Yes | There was no evidence of non-coherence between data sources, collection, analysis and interpretation. |

**Kimzey, Mastel-Smith et al. 2016 (63)**

| Methods | Three groups pre- and post-tests | | |
| --- | --- | --- | --- |
| Participants | Nursing students | | |
| Setting | Community health faculty | | |
| Country | USA | | |
| Intervention | - Alzheimer’s disease online module - Engagement with people with AD at a memory care unit and dementia day centre | | |
| Comparator | Control group no dementia-specific intervention | | |
| Outcomes | Knowledge  Attitudes | | |
| Educational Content | - Cognitive assessment - Person-centred care - Changes in thinking as dementia progresses - How changes impact behaviour - How to understand behaviour as communication | | |
| Pedagogical Approach | Online learning - not otherwise specified. | | |
| Technological Specifications | Online – not otherwise specified. | | |
| Educational Theory | Kolb's experiential learning theory (pertains to comparator group). | | |
|  | | | |
| **Quality Criteria (MMAT 3)** | | **Author Judgement** | **Support for Judgement** |
| Are the participants representative of the target population? | | Can’t tell | The authors acknowledged limitations of the convenience sample. |
| Are measurements appropriate regarding both the outcome and intervention (or exposure)? | | Can’t tell | There was no evidence of validity or reliability for outcome measures specific to this study. |
| Are there complete outcome data? | | Yes | Missing survey data (6%) was accounted for with additional missing data for 3% of participants (ADKS) and is satisfactory based on the arbitrary threshold (20%). However, differences in students enrolled (100) and intervention group and control participants (101) were noted. |
| Are the confounders accounted for in the design and analysis? | | No | Potential confounders were identified with no significant differences between groups. Intervention(s) duration was not reported with potential for confounders (maturation effects) between pre- and post tests. |
| During the study period, is the intervention administered (or exposure occurred) as intended? | | Can’t tell | There was lack of assurances re intervention controls including place of online module completion and variations in clinical group experiences. |
| **Quality Criteria (MMAT 1)** | | **Author Judgement** | **Support for Judgement** |
| Is the qualitative approach appropriate to answer the research question? | | Yes | Focus groups with content analysis. |
| Are the qualitative data collection methods adequate to address the research question? | | Yes | Semi-structured interviews from audiotaped focus group. |
| Are the findings adequately derived from the data? | | Yes | Content analysis including line by line coding and categorized codes. Themes emerged from the narrative texts. Constant comparison method used throughout analysis. Second coder interpretation and audit trail developed. |
| Is the interpretation of results sufficiently substantiated by data? | | Yes | Themes were presented and quotations justified themes. |
| Is there coherence between qualitative data sources, collection, analysis and interpretation? | | Yes | There was no evidence of non-coherence between data sources, collection, analysis and interpretation. |

**Luconi 2008 (64)**

| Methods | Single group pre- and post-test (with follow up) | | |
| --- | --- | --- | --- |
| Participants | Family physicians | | |
| Setting | Primary care (rural) | | |
| Country | Canada | | |
| Intervention | Early Alzheimer’s Disease (AD) Program: Web-based Continuing Medical Education Program | | |
| Comparator | Not applicable | | |
| Outcomes | Knowledge | | |
| Educational Content | Learning outcomes were to increase rural family physician’s knowledge, skills, and confidence in:   - Diagnosing the early symptoms of AD - Prescribing appropriate and safe treatment for early and moderate stages of AD - Managing the care of patients and their caregivers during the early stages of AD | | |
| Pedagogical Approach | Web-based resource using mini-lectures and a case study approach. Individual and collaborative activities include asynchronous discussions (individual, paired, and moderated plenary program activities), quizzes (check out quiz ensure selective release of subsequent modules), and hypertext links to resources. | | |
| Technological Specifications | Designed and implemented on Web CT. Basic hardware and software required are computer with internet access, a modem (56K or higher) or cable, and a web browser (Explorer 4 and up, or Netscape 6). | | |
| Educational Theory | Constructivism:  • Cognitive constructivism  • Socio-constructivism  Four-stage theory of physician learning (based on social constructivism)  • Includes reference to Maslow’s theory of self-actualisation  Clinical reasoning models | | |
|  | | | |
| **Quality Criteria (MMAT 3)** | | **Author Judgement** | **Support for Judgement** |
| Are the participants representative of the target population? | | Can’t tell | Convenience sample. |
| Are measurements appropriate regarding both the outcome and intervention (or exposure)? | | Can’t tell | There was evidence of content validity of the objective measures (MCQ). Reliability was not reported. |
| Are there complete outcome data? | | Yes | All participants completed all outcome measures. |
| Are the confounders accounted for in the design and analysis? | | No | Confounders were not accounted for with significant duration between pre- and post-tests (potential maturation effects). |
| During the study period, is the intervention administered (or exposure occurred) as intended? | | Yes | There was no evidence that the intervention was not administered as intended during the intervention period. |

Note: Luconi (2008) is a comprehensive descriptive case study. The quality assessment provided relates to the pre- and post-test objectives measures included within the study and is not intended as an appraisal of the complete work.

**Matsumura, Shinno et al. 2018 (75)**

| Methods | Equivalent control group pre- and post-test | | |
| --- | --- | --- | --- |
| Participants | Medical students | | |
| Setting | University | | |
| Country | Japan | | |
| Intervention | Clinical simulator with virtual patients (VP) and conventional learning | | |
| Comparator | Conventional learning | | |
| Outcomes | Satisfaction  Knowledge | | |
| Educational Content | Simulated learning in practice concept with VPs with dementia:   - 84-year-old woman with dementia of Alzheimer type - 73-year-old woman with dementia with Lewy bodies - 77-year-old man with frontotemporal lobar degeneration - 73-year-old man with vascular dementia   The learner can interview patients and their families, read their facial expressions during the interview, run tests, give diagnoses, and give prescriptions; patients and their families can ask questions about the disease from which the patient is suffering, the tests that the patient may undergo, and the prescriptions that the patient may receive; learners can respond to these questions; and orders for tests and prescriptions can be conducted using a realistic electronic medical record system. | | |
| Pedagogical Approach | VP case study approach. Responses to patient questions using multiple choice approach, ordering diagnostic tests, giving prescriptions etc. with immediate virtual feedback or human interaction where necessary. When the students completed some form of action (examination, diagnosis, explanation, prescription, etc.), a virtual doctor appeared and gave advice. The virtual doctor provided a commentary, which included an indication of whether the student’s behaviour was correct. Human (teacher) interaction available if necessary. | | |
| Technological Specifications | Simulator can run in an internet browser or as a standalone system. The software ran on Windows 7 operating system using Internet Explorer 9.010 browser and Adobe Flash Player 18.0.0.194. | | |
| Educational Theory | None specified. | | |
|  | | | |
| **Quality Criteria (MMAT 3)** | | **Author Judgement** | **Support for Judgement** |
| Are the participants representative of the target population? | | Can’t tell | The sampling approach was not clearly described. |
| Are measurements appropriate regarding both the outcome and intervention (or exposure)? | | Can’t tell | Content validation and reliability testing were reported for the knowledge test only and not the ARCS. |
| Are there complete outcome data? | | Yes | All students participated. |
| Are the confounders accounted for in the design and analysis? | | No | There were attempts to mitigate confounding effects between groups; however, there was significant time and opportunity for confounders between pre- and post knowledge tests. |
| During the study period, is the intervention administered (or exposure occurred) as intended? | | Yes | There was no evidence that the intervention was not administered as planned. |

**Rababa, Masha'al 2020 (67)**

| Methods | Equivalent control group pre- and post-test | | |
| --- | --- | --- | --- |
| Participants | Nursing students | | |
| Setting | University of Science and Technology / College of Nursing | | |
| Country | Jordan | | |
| Intervention | Computer-based branching path simulation (BPS) for pain management in people with dementia (and presentations and discussions using case scenarios / vignettes) | | |
| Comparator | Traditional learning (presentations and discussions using case scenarios / vignettes) | | |
| Outcomes | Skills (critical thinking) | | |
| Educational Content | BPS is an interactive learning method using case scenarios that guide the learner though a step by step decision making process. BPS gives learners the opportunity to make decisions according to their level of skills and knowledge and get feedback immediately which help them to demonstrate critical thinking skills in a safe and supported environment before dealing with complex and real-life case scenarios.  Example case scenario / vignette:  *“A 96-year-old person with severe dementia, a female on one day was screaming and crying during the morning care. She had been in the nursing home for 23 months, was non-ambulatory, had communication deficit (aphasia), and she had bilateral ankle and knee pain. In addition to crying, she consistently screamed ‘oooh, oooh, oooh,’when changed her position, she started to be non-cooperative to the care and looked very sad and depressed when approached. The nurse interpreted these odd behaviours as anxiety and responded by providing her care slowly with other nurses' help”* | | |
| Pedagogical Approach | BPS as above. | | |
| Technological Specifications | Electronically produced BPS – not otherwise specified. | | |
| Educational Theory | BPS is informed by immediate feedback and analytic decision-making learning theories and the principles of behaviourism and cognitivism. | | |
|  | | | |
| **Quality Criteria (MMAT 3)** | | **Author Judgement** | **Support for Judgement** |
| Are the participants representative of the target population? | | Can’t tell | The authors reported random assignment to treatment/non-treatment groups from a convenience sample. The process of randomisation was not stipulated. |
| Are measurements appropriate regarding both the outcome and intervention (or exposure)? | | Yes | There was evidence of content validity and evidence of reliability testing in context. |
| Are there complete outcome data? | | Can’t tell | The authors reported outcome data for 102 participants; however, demographic data alluded to 104 participants. |
| Are the confounders accounted for in the design and analysis? | | No | Some potential confounders were identified and are not included in analysis. |
| During the study period, is the intervention administered (or exposure occurred) as intended? | | Yes | There was no evidence that the intervention did not occur as intended. |

Note: BPS is not at technology-enabled dementia education intervention per se. However, the purpose of this study was to examine the efficacy of a computer-based (electronically-produced) BPS to improve the critical thinking skills of nursing students about pain assessment and management in people with dementia and was considered relevant for inclusion into the review on this basis.

**Ruiz, Smith et al. 2006 (66)**

| Methods | Single group pre- and post-tests | | |
| --- | --- | --- | --- |
| Participants | Licensed practical nursing students | | |
| Setting | College medical campus | | |
| Country | USA | | |
| Intervention | Multimedia training CD-ROM: Alzheimer’s and other Dementias | | |
| Comparator | Not applicable | | |
| Outcomes | Knowledge  Skills  Attitudes | | |
| Educational Content | - Understanding Dementia: This module presents the definition of dementia and describes the cognitive and functional changes seen as dementia progresses - Communication: Learners are shown the changes that occur in communication as dementia progresses. Verbal and non-verbal types of communication are discussed. Effective communication strategies are demonstrated - Distress Behaviours: Learners are shown how to recognize, prevent, and manage distress behaviours. The triggers and consequences of common distress behaviours are discussed - Loved Ones: This module emphasized the importance of involving family members and friends in the care of the resident with dementia. Family emotions and stress as well as strategies for helping them cope are addressed - Activities of Daily Living: This module stresses the importance of promoting independence in activities of daily living and offers specific strategies to maximize independence as dementia progresses | | |
| Pedagogical Approach | Computerised learning using text, animations, video, audio, and interactive exercises.   - Modules follow a similar format, beginning with an introduction to the topic, then a presentation of types of issues often encountered, followed by a presentation of solutions. Each module ends with a set of practice exercises | | |
| Technological Specifications | CD-ROM for use on individual computers. | | |
| Educational Theory | None specified. | | |
|  | | | |
| **Quality Criteria (MMAT 3)** | | **Author Judgement** | **Support for Judgement** |
| Are the participants representative of the target population? | | Can’t tell | There was insufficient information to judge representativeness. |
| Are measurements appropriate regarding both the outcome and intervention (or exposure)? | | Can’t tell | There was no evidence of validity or reliability of the outcome measures. |
| Are there complete outcome data? | | Can’t tell | 38 LPNs took part the study. It was not made clear if all participants completed pre- and post-tests. |
| Are the confounders accounted for in the design and analysis? | | Yes | Some potential confounders were identified and considered in the analysis. Testing was immediately before and after the 4-hour training. |
| During the study period, is the intervention administered (or exposure occurred) as intended? | | Yes | There was no evidence that the intervention was not administered as planned. |

**Tomaz, Jose Batista Cisne, Mamede et al. 2015 (76)**

| Methods | Equivalent control group pre- and post-test | | |
| --- | --- | --- | --- |
| Participants | Family physicians | | |
| Setting | Family health teams | | |
| Country | Brazil | | |
| Intervention | Online problem-based learning: Clinical Approach for Elderly with Dementia | | |
| Comparator | Non-participation in online problem-based learning | | |
| Outcomes | Knowledge  Skills | | |
| Educational Content | Clinical approach for elderly with dementia - not otherwise specified – includes contextually relevant clinical problems posed to the learning group. A course guide was provided. | | |
| Pedagogical Approach | Online problem-based learning involving virtual tutorial groups supervised by a facilitator using asynchronous virtual forums and synchronous chat using a three-phase process:   - analysis - individual study - problem solving   Complementary educational strategies included clinical skills training, team and individual projects and community practice.  The course is 120 hours which includes 20 hours face-to-face instruction.  Online learning tools included video-lectures, CD-ROM, and texts. | | |
| Technological Specifications | Online learning provided in learning management system (MOODLE). | | |
| Educational Theory | Problem based learning is a well-established constructivist educational approach. | | |
|  | | | |
| **Quality Criteria (MMAT 3)** | | **Author Judgement** | **Support for Judgement** |
| Are the participants representative of the target population? | | Can’t tell | The sampling approach was not described. |
| Are measurements appropriate regarding both the outcome and intervention (or exposure)? | | Yes | The knowledge test had borderline reliability otherwise all measurements were appropriate with evidence of validity and reliability. |
| Are there complete outcome data? | | Yes | Non-participation was less than 20% (17%) and is acceptable based on this arbitrary threshold. Reasons for nonparticipation were provided. |
| Are the confounders accounted for in the design and analysis? | | No | Confounders were not accounted with potential for temporal change from pre- to post-tests. |
| During the study period, is the intervention administered (or exposure occurred) as intended? | | Can’t tell | It was not clear if additional face-to-face skills training by the experimental group was intended. |

**Tsai, Kitch et al. 2018 (77)**

| Methods | Equivalent control group pre- and post-test | | |
| --- | --- | --- | --- |
| Participants | Certified nursing assistants and nursing home residents | | |
| Setting | Long term care | | |
| Country | USA | | |
| Intervention | Computer-based simulation (level of dressing assistance for people with dementia) and face-to-face training module | | |
| Comparator | Level of assistance face-to-face training module | | |
| Outcomes | Behaviours  Results | | |
| Educational Content | The simulation involves an elderly women with moderate dementia who requires assistance with dressing through a series of progressive tasks that require variable levels of assistance:   - verbal prompt - gesture/modelling - physical prompt - occasional physical guidance - complete physical guidance | | |
| Pedagogical Approach | Simulated learning activity where the learner chooses the appropriate level of assistance required for specific dressing tasks at basic and advanced levels. The simulator uses video clips of an elder actor to initiate dressing processes. The elder actor will not perform tasks until the correct level of assistance is selected. If the user provides inappropriate (too little or too much) assistance, the elder responds with passive noncompliance or agitation. The goal is to minimize agitation, maximize independence, and enable dressing with minimal decision-making delays. | | |
| Technological Specifications | Computer-based simulation on tablet device. | | |
| Educational Theory | Banduras social learning theory / Theory of planned behaviour. | | |
|  | | | |
| **Quality Criteria (MMAT 3)** | | **Author Judgement** | **Support for Judgement** |
| Are the participants representative of the target population? | | Can’t tell | The sampling approach was not clear. |
| Are measurements appropriate regarding both the outcome and intervention (or exposure)? | | Yes | The BPDS was content validated previously (no obvious requirement for validation in context) and inter-rater reliability testing was described. |
| Are there complete outcome data? | | No | Data were analysed for 9 out of 12 eligible dyads. The missing data is 25% and more than the arbitrary threshold (20%). |
| Are the confounders accounted for in the design and analysis? | | No | Confounders were identified with no significant differences between groups. Effects of temporal change were not accounted for. |
| During the study period, is the intervention administered (or exposure occurred) as intended? | | Yes | There was no evidence that the intervention was not administered as intended. |

**Vollmar, Mayer et al. 2010 (69)**

| Methods | Cluster randomised trial | | |
| --- | --- | --- | --- |
| Participants | General Practitioners | | |
| Setting | Primary Care / GP quality circles | | |
| Country | Germany | | |
| Intervention | Blended learning (presentation of a dementia guideline in online modules and a structured discussion during a quality circle meeting) | | |
| Comparator | Presentation of dementia guideline in a lecture and a structured discussion during a quality circle meeting   - Additional (non-randomised) control group of participants who received a printed pocket version of the dementia guideline | | |
| Outcomes | Knowledge | | |
| Educational Content | - Two interactive case stories on dementia related to the guideline content (diagnosis or management and therapy of dementia) - Three testing modules allowing acquisition of CME credit points. They covered the same topics as the interactive case stories (as well as the lecture) - The dementia guideline was provided (diagnosis and therapy of dementia - exact content not available) | | |
| Pedagogical Approach | Blended learning approach at GP quality circles using online learning with interactive case studies, tests, and printed material. | | |
| Technological Specifications | Online learning – not otherwise specified. | | |
| Educational Theory | None specified. | | |
|  | | | |
| **Quality Criteria (MMAT 2)** | | **Author Judgement** | **Support for Judgement** |
| Is randomization appropriately performed? | | Yes | Stratified randomisation was at cluster level by a statistician. Group allocation was concealed appropriately. |
| Are the groups comparable at baseline? | | Can’t tell | Cluster and participant level baseline data were available and were generally comparable. It was not clear if similarities / differences were significant. |
| Are there complete outcome data? | | No | There was missing data from 65% of participants (GPs) from all groups between t_0_ (first knowledge test and consent) and t_2_. Data was missing from 46% of participants from intervention groups between t_0_ (first knowledge test and consent) and t_1_. Most data was lost to follow up. In both scenarios, the amount of missing data was higher than the arbitrary threshold (20%). |
| Are outcome assessors blinded to the intervention provided? | | Can’t tell | There was no information on outcome assessors and limited information on assessment processes. |
| Did the participants adhere to the assigned intervention? | | No | There was a high proportion of ‘non-users’ identified from study group A and variation with engagement amongst users. |

**Westmoreland, Counsell et al. 2010 (70)**

| Methods | Randomised controlled trial | | |
| --- | --- | --- | --- |
| Participants | Medical residents | | |
| Setting | University (school of medicine) | | |
| Country | USA | | |
| Intervention | Web-based training in geriatrics (includes a dementia education module) | | |
| Comparator | Paper-based learning group | | |
| Outcomes | Knowledge  Behaviours | | |
| Educational Content | Web-based module contained eight sections:   - Learning objectives - Overview - Differential diagnosis - Diagnostic - Work-up - Treatment - Summary - Glossary of Terms References Evaluation | | |
| Pedagogical Approach | Web-based training using case-based instruction. Modules were textual, with pictorial content. Video streaming was included that demonstrated how to administer the Mini-Mental State Examination (dementia module). | | |
| Technological Specifications | Web-based learning on ‘A New Global Environment for Learning’ (ANGEL) curriculum repository. | | |
| Educational Theory | None specified. | | |
|  | | | |
| **Quality Criteria (MMAT 2)** | | **Author Judgement** | **Support for Judgement** |
| Is randomization appropriately performed? | | Can’t tell | Participants were randomised using a block format based on ambulatory rotations. It was not clear if it was possible to predict allocations. |
| Are the groups comparable at baseline? | | Yes | Group sizes were equal at baseline and the authors reported that there were no significant differences in baseline characteristics. |
| Are there complete outcome data? | | No | There was substantial variability in the completeness of the outcome data across measures. |
| Are outcome assessors blinded to the intervention provided? | | Can’t tell | It was unclear how the pre and post paper tests were completed and assessed. It was not clear if the research assistant was blinded when completing/scoring the encounter checklists. It was not clear if the PI was blinded when reviewing EMRS orders. |
| Did the participants adhere to the assigned intervention? | | No | The study ended one month early because of resident  and attending physician dissatisfaction. |
