# Supplementary material for: The characteristics of effective technology-enabled dementia education: a systematic review and mixed research synthesis
Source: Syst Rev. 2022 Feb 23;11:34. doi: 10.1186/s13643-021-01866-4 (PMC8865181; doi:10.1186/s13643-021-01866-4)
Supplement: Supplementary file 5 — Additional file 5. [file 13643_2021_1866_MOESM5_ESM.docx]

**Additional file 5. Excluded Studies**

| **Narrative and Survey Evaluations** | | | |
| --- | --- | --- | --- |
| **Citation** | **Title** | **Rationale** | **Search Date** |
| (Ball, Bluteau et al. 2015) | MyShoes: An immersive simulation of dementia. | Describes the development of an immersive simulation of dementia and includes narrative feedback from health and social care students. | 2018 |
| (Bonnel, Fletcher et al. 2007) | Integrating geriatric resources into the classroom: A virtual tour example. | Describes a ‘virtual tour’ for Alzheimer’s Disease education with brief evaluation by nursing students in narrative data (fast feedback). | 2018 |
| (Bryan, Asghar-Ali 2020) | Development and dissemination of an interprofessional online dementia training curriculum. | Evaluation of an online dementia education program in rural areas. Survey (post-test only) and narrative data. | 2020 |
| (Canty, Goldberg et al. 2015) | Meeting the challenge of designing and delivering an entry level unit of study to engage and inspire adult learners in online neuroscience education in a bachelor of dementia care. | Describes a bachelor of dementia care degree program. Evaluation of a program foundation module by adult learners. Survey and narrative data. | 2018 |
| (Davison, Housden et al. 2019) | Using interprofessional dementia learning opportunities to prepare the future healthcare workforce: findings from a pilot study. | Evaluation of a dementia awareness training program delivered online or face-to-face. Evaluation by health care students with survey data (limited ‘online’ evaluation data). | 2020 |
| (DeSouza, Pit et al. 2020) | Translating facilitated multimodal online learning into effective person-centred practice for the person living with dementia among health care staff in Australia: an observational study. | Evaluation of an online dementia education program for health care professionals from cross sectional analysis of survey data. | 2020 |
| (Dobbs, Hobday et al. 2018) | Certified nursing assistants' perspectives of the CARES activities of daily living dementia care program. | Evaluation of an online dementia care program by nursing assistants. Survey and narrative data. | 2018 |
| (George, Dellasega 2011) | Use of social media in graduate-level medical humanities education: Two pilot studies from Penn State College of Medicine. | Medical students evaluate the integration of social media into a dementia education module. Brief survey and narrative data. | 2018 |
| (Goldberg, Carr et al. 2016}) | Making neuroscience important and relevant: Online learning in an innovative bachelor of dementia care program. | Evaluation of four neuroscience units in a bachelor of dementia care program. Includes the effects of previous university experience on students’ grades and learner evaluations with narrative data. | 2018 |
| (Innes, Mackay et al. 2006) | Dementia studies online: Reflections on the opportunities and drawbacks of eLearning. | Describes opportunities and drawbacks of a postgraduate dementia studies eLearning program for health and social care practitioners (from author experience and student feedback). | 2018 |
| (Innes, Kelly et al. 2012) | An evaluation of an online postgraduate dementia studies program. Gerontology & Geriatrics Education. | Describes an online postgraduate dementia education program. Evaluation by adult learners. Survey and narrative data. | 2018 |
| (Jennings, Boyle et al. 2018) | The development and evaluation of an online dementia resource for primary care-based health professionals. | Describes the development of an online dementia resource for primary care professionals. Evaluation by potential end-users. Brief survey and narrative data. | 2018 |
| (Kovacich, Garrett et al. 2006) | New learning programs in cognitive vitality, Alzheimer's disease, and related dementias. | Describes a web-based dementia education program – “Alzheimer’s Disease and Related Dementias”. Multidisciplinary evaluation using survey data. | 2018 |
| (MacDonald, Stodel et al. 2006) | Online dementia care training for healthcare teams in continuing and long-term care homes: a viable solution for improving quality of care and quality of life for residents. | Describes the design and development of an online dementia care program using mixed methods. Health care workers and professionals evaluate the program. Survey and narrative data. | 2018 |
| (Pulman, Galvin et al. 2012) | Empathy and dignity through technology: Using lifeworld-led multimedia to enhance learning about the head, heart and hand. | Describes a web-based case study on the human experience of living with dementia. Health and social care students provide narrative evaluations. | 2018 |
| (Tomaz, van et al. 2013) | The design and program evaluation of a distributed PBL curriculum for training family doctors in Brazil. | Evaluation of an online problem-based learning curriculum by family doctors. Survey and narrative data. | 2018 |

| **Not a TEDE Intervention** | | | |
| --- | --- | --- | --- |
| **Citation** | **Title** | **Rationale** | **Search Date** |
| (Banks, Waugh et al. 2014) | Enriching the care of patients with dementia in acute settings? The dementia champions programme in Scotland. | Evaluates a multicomponent dementia training program with insufficient TEDE specific outcome data. | 2018 |
| (Bánszki, Beilby et al. 2018) | A clinical educator's experience using a virtual patient to teach communication and interpersonal skills. | Explores a clinical educators experience of using a simulated patient in a virtual learning environment to teach communication and interpersonal skills to speech and language therapy students. | 2018 |
| (Calleson, Sloane et al. 2006) | Effectiveness of mailing 'bathing without a battle' to all US nursing homes. Gerontology & Geriatric Education. | Describes the dissemination of an educational intervention (CD-ROM *or* video) to educate nursing home staff on techniques to reduce agitation and aggression during bathing. Some evaluative data is available with limited discernment between CD-ROM or video related outcomes. | 2018 |
| (Cotter, Hasan et al. 2019) | A Practice Improvement Project to Increase Advance Care Planning in a Dementia Specialty Practice. | Evaluates the effectiveness of a face-to-face *or* online training intervention to improve physician knowledge of advance care planning in dementia. TEDE specific outcome data not available. | 2020 |
| (Detroyer, Dobbels et al. 2016) | The effect of an interactive delirium e-learning tool on healthcare workers' delirium recognition, knowledge and strain in caring for delirious patients: A pilot pre-test/post-test study. | Evaluates the effectiveness of an e-learning intervention for delirium. | 2018 |
| (Giguere, Lawani et al. 2018) | Tailoring and evaluating an intervention to improve shared decision-making among seniors with dementia, their caregivers, and healthcare providers: study protocol for a randomized controlled trial. | Describes a study protocol to evaluate a multicomponent shared decision-making intervention for people with dementia, families, and professionals. | 2020 |
| (McCrow, Sullivan et al. 2014) | Delirium knowledge and recognition: A randomized controlled trial of a web-based educational intervention for acute care nurses. | Evaluates the effectiveness of a delirium educational website. | 2018 |
| (Muñoz-Narbona, Roldán-Merino et al. 2019) | Impact of a Training Intervention on the Pain Assessment in Advanced Dementia (PAINAD) Scale in Noncommunicative Inpatients. | Evaluates the impact of an online nurse training intervention on pain management in non-communicative patients (based on a tool to assess pain in advanced dementia). | 2020 |
| (O'Connell, Guse et al. 2018) | Does restructuring theory and clinical courses better prepare nursing students to manage residents with challenging behaviors in long-term care settings? | Evaluates nursing students’ preparedness for responsive behaviors following theory / course restructuring which includes an online learning module for managing behaviors of concern. | 2018 |
| (Sehgal, Syed et al. 2019) | Introducing Aquifer Geriatrics, the American Geriatrics Society National Online Curriculum. | Describes the development of an online curriculum for generic geriatric education. | 2020 |
| (Surr, Sass et al. 2019) | A collective case study of the features of impactful dementia training for care home staff. | Investigates features associated with effective dementia training for care home staff. | 2020 |
| (Van Mierlo, Meiland et al. 2015) | Evaluation of DEM-DISC, customized e-advice on health and social support services for informal carers and case managers of people with dementia; a cluster randomized trial. | Evaluates an intervention that provides information on health / social care services for people with dementia and their carers. | 2018 |

TEDE (Technology-enabled dementia education)

| **Not Relevant to Review Outcomes** | | | |
| --- | --- | --- | --- |
| **Citation** | **Title** | **Rationale** | **Search Date** |
| (Cartwright, Franklin et al. 2015) | Promoting collaborative dementia care via online interprofessional education. | Evaluates the effectiveness of an online interprofessional education program using a dementia case study to improve interprofessional socialization and values. | 2018 |
| (Degryse, De Lepeleire et al. 2009) | An evaluation of a computer-based education program for the diagnosis and management of dementia in primary care. An international study of the transcultural adaptations necessary for European dissemination. | Describes adaptations of a computer-based dementia education program for use in different European countries. | 2018 |
| (King, Kelder et al. 2013) | Something for everyone: MOOC design for informing dementia education and research. | Describes the design of a dementia MOOC with limited (pilot) study evaluation data. | 2018 |
| (Llambi, Margolis et al. 2008) | Distance education for physicians: Adaptation of a Canadian experience to Uruguay. | Describes the process of adapting a course on Alzheimer’s Disease from the Canadian context for online use in Uruguay. | 2018 |
| (MacDonald, Stodel, Casimiro, and Weaver 2006) | Using community-based participatory research for an online dementia care program. | Describes ‘community-based participatory research’ relating to the design and evaluation of an online dementia education program. | 2018 |
| (McInerney, Doherty et al. 2018) | How is palliative care understood in the context of dementia? results from a massive open online course. | Explores the meaning of palliative care (in the context of dementia) from participant contributions to a dementia MOOC forum. | 2018 |
| (Robertshaw, Babicova 2019) | Discovering the memory thief: MOOC participants' personal experiences of dementia. | Explores experiences of dementia among MOOC participants. Does not evaluate MOOC effectiveness. | 2020 |
| (Robertshaw, Cross 2019a) | Experiences of Integrated Care for Dementia from Family and Carer Perspectives: A Framework Analysis of Massive Open Online Course Discussion Board Posts. | Explores views of integrated health and social care for dementia from the perspectives of dementia MOOC participants. Does not evaluate MOOC effectiveness. | 2020 |
| (Robertshaw, Cross 2019b) | Roles and responsibilities in integrated care for dementia. Journal of Integrated Care. | Explores roles / responsibilities in relation to integrated care from the prespecifies of dementia MOOC participants. Does not evaluate MOOC effectiveness. | 2020 |
| (Waldorff, Steenstrup et al. 2008) | Diffusion of an e-learning programme among Danish general practitioners: A nation-wide prospective survey. | Examines the uptake of a dementia e-learning program for Danish general practitioners. Some (limited) evaluation data included. | 2018 |

MOOC (massive open online course)

| **Includes Non-Professional Participants** | | | |
| --- | --- | --- | --- |
| **Citation** | **Title** | **Rationale** | **Search Date** |
| (Dassel, Butler et al. 2020) | Development and evaluation of Alzheimer's Disease and Related Dementias (ADRD) best care practices in long-term care online training program. | Describes and evaluates online training for Alzheimer’s Disease and related dementias for professional and informal carers of people with dementia. Professional roles not specified in outcome data. | 2020 |
| (Eccleston, Doherty et al. 2019) | Building dementia knowledge globally through the Understanding Dementia Massive Open Online Course (MOOC). | Evaluates the effectiveness of a dementia MOOC in educating people about dementia among participants with work, family, or educational exposure to dementia. Professional roles not specified in outcome data. | 2020 |
| (Gitlin, Hodgson 2016) | Online training--can it prepare an eldercare workforce? | Literature review and description of a dementia MOOC for professionals and non-professionals with brief evaluation (participant testimonies). | 2018 |
| (Hattink, Meiland et al. 2015) | Web-based STAR E-learning course increases empathy and understanding in dementia caregivers: results from a randomized controlled trial in the Netherlands and the United Kingdom. | Evaluates a dementia e-learning resource for informal carers, volunteers, and professional carers. Professional roles not specified. | 2018 |
| (Petronzi, Hadi 2016) | Exploring the factors associated with MOOC engagement, retention and the wider benefits for learners. | Explores the benefits of a dementia MOOC from professional and non-professional learner perspectives. Professional roles not consistently specified in outcome data. | 2018 |
| (Pleasant, Molinari et al. 2017) | An evaluation of the CARES® dementia basics program among caregivers. | Evaluates a dementia online training program for formal and informal caregivers. Professional roles not specified in outcome data and differential analysis not performed based on formal / informal carer role. | 2018 |
| (Pleasant 2018) | Improving dementia care through online training programs: A systematic review and evaluation. | Evaluates an online dementia education program among informal and formal caregivers. Professional roles not explicitly specified in outcome data. | 2018 |

MOOC (massive open online course)

**Reference List of Excluded Studies**

Ball S, Bluteau P, Clouder D, et al. MyShoes: An immersive simulation of dementia. Proceedings of the International Conference on e-Learning, ICEL. 2015; 16-23.

Banks P, Waugh A, Henderson J, et al. Enriching the care of patients with dementia in acute settings? The dementia champions programme in Scotland. Dementia. 2014; 13(6):717-736.

Bánszki F, Beilby J, Quail M, et al. A clinical educator's experience using a virtual patient to teach communication and interpersonal skills. Australasian Journal of Educational Technology. 2018; 34(3):60-73.

Bonnel W, Fletcher K, Wingate A. Integrating geriatric resources into the classroom: A virtual tour example. Geriatric Nursing. 2007; 28(5):301-305.

Bryan J & Asghar-Ali A. Development and dissemination of an interprofessional online dementia training curriculum. Journal of the American Geriatrics Society. 2020; 68(1):192-197.

Calleson D, Sloane P, Cohen L. Effectiveness of mailing 'bathing without a battle' to all US nursing homes. Gerontology & Geriatric Education. 2006; 27(1):67-79.

Canty A, Goldberg L, Ziebell J, et al. Meeting the challenge of designing and delivering an entry level unit of study to engage and inspire adult learners in online neuroscience education in a bachelor of dementia care. Proceedings of the International Conference of Education, Research and Innovation ICERI. 2015; 3941-3951.

Cartwright J, Franklin D, Forman D, et al. Promoting collaborative dementia care via online interprofessional education. Australasian Journal on Ageing. 2015; 34(2):88-94.

Cotter V, Hasan M, Ahn J, et al. A Practice Improvement Project to Increase Advance Care Planning in a Dementia Specialty Practice. American Journal of Hospice & Palliative Medicine. 2019; 36(9):831-835.

Dassel K, Butler J, Telonidis J, et al. Development and evaluation of Alzheimer's Disease and Related Dementias (ADRD) best care practices in long-term care online training program. Educational Gerontology. 2020; 46(3):150-157.

Davison E, Housden S, Lindqvist S. Using interprofessional dementia learning opportunities to prepare the future healthcare workforce: findings from a pilot study. Journal of Interprofessional Care. 2019; 33(6):816-819.

Degryse J, De Lepeleire J, Southgate L, et al. An evaluation of a computer-based education program for the diagnosis and management of dementia in primary care. An international study of the transcultural adaptations necessary for European dissemination. Medical Teacher. 2009; 31(5):397-402.

Desouza K, Pit S, Moehead A. Translating facilitated multimodal online learning into effective person-centred practice for the person living with dementia among health care staff in Australia: an observational study. BMC Geriatrics. 2020; 20:33

Detroyer E, Dobbels F, Debonnaire D, et al. The effect of an interactive delirium e-learning tool on healthcare workers' delirium recognition, knowledge and strain in caring for delirious patients: A pilot pre-test/post-test study. BMC Medical Education. 2016; 16:17.

Dobbs D, Hobday J, Roker R, et al. Certified nursing assistants' perspectives of the CARES activities of daily living dementia care program. Applied Nursing Research. 2018; 39:244-248.

Eccleston C, Doherty K, Bindoff A, et al. Building dementia knowledge globally through the Understanding Dementia Massive Open Online Course (MOOC). NPJ Science of Learning. 2019; 3.

George D & Dellasega C. Use of social media in graduate-level medical humanities education: Two pilot studies from Penn State College of Medicine. Medical Teacher. 2011; 33(8):e429-e434.

Giguere A, Lawani M, Fortier-Brochu É, et al. Tailoring and evaluating an intervention to improve shared decision-making among seniors with dementia, their caregivers, and healthcare providers: study protocol for a randomized controlled trial. Trials. 2018; 19:332.

Gitlin L & Hodgson N. Online training--can it prepare an eldercare workforce? Generations. 2016; 40(1):71-81.

Goldberg L, Carr A, Canty A, et al. Making neuroscience important and relevant: Online learning in an innovative bachelor of dementia care program. In: Vincenti G., Bucciero A., Vaz de Carvalho C. (eds) E-Learning, E-Education, and Online Training. eLEOT 2015. Lecture Notes of the Institute for Computer Sciences, Social Informatics and Telecommunications Engineering, 2016; 160:84-91.

Hattink B, Meiland F, van der Roest H, et al. Web-based STAR E-learning course increases empathy and understanding in dementia caregivers: results from a randomized controlled trial in the Netherlands and the United Kingdom. Journal of Medical Internet Research. 2015; 17(10):e241.

Innes A, Kelly F, McCabe L. An evaluation of an online postgraduate dementia studies program. Gerontology & Geriatrics Education. 2012; 33(4):364-382.

Innes A, Mackay K, McCabe L. Dementia studies online: Reflections on the opportunities and drawbacks of eLearning. Journal of Vocational Education and Training. 2006; 58(3):303-317.

Jennings A, Boyle S, Foley T. The development and evaluation of an online dementia resource for primary care based health professionals. Internet Interventions. 2018; 11:47-52.

King C, Kelder J, Phillips R, et al. Something for everyone: MOOC design for informing dementia education and research. Proceedings of the European Conference on e-Learning, ECEL. 2013; 191-198.

Kovacich J, Garrett R, Forti E. New learning programs in cognitive vitality, Alzheimer's disease, and related dementias. Gerontology & Geriatrics Education. 2006; 26(4):47-61.

Llambi L, Margolis A, Toews J, et al. Distance education for physicians: Adaptation of a Canadian experience to Uruguay. Journal of Continuing Education in the Health Professionals. 2008; 28(2):79-85.

MacDonald C, Stodel E, Casimiro L. Online dementia care training for healthcare teams in continuing and long-term care homes: a viable solution for improving quality of care and quality of life for residents. International Journal on E-Learning. 2006; 5(3):373-399.

MacDonald C, Stodel E, Casimiro L, et al. Using community-based participatory research for an online dementia care program. Canadian Journal of Program Evaluation. 2006; 21(2):81-104.

McCrow J, Sullivan K, Beattie E. Delirium knowledge and recognition: A randomized controlled trial of a web-based educational intervention for acute care nurses. Nurse Education Today. 2014; 34(6):912-917.

McInerney F, Doherty K, Bindoff A, et al. How is palliative care understood in the context of dementia? results from a massive open online course. Palliative Medicine. 2018; 32(3):594-602.

Muñoz-Narbona L, Roldán-Merino J, Lluch-Canut T, et al. Impact of a Training Intervention on the Pain Assessment in Advanced Dementia (PAINAD) Scale in Noncommunicative Inpatients. Pain Management Nursing 2019; 20(5):468-474.

O'Connell B, Guse L, Greenslade L. Does restructuring theory and clinical courses better prepare nursing students to manage residents with challenging behaviors in long-term care settings? Gerontology & Geriatrics Education. 2018; 1-15.

Petronzi D & Hadi M. Exploring the factors associated with MOOC engagement, retention and the wider benefits for learners. European Journal of Open, Distance and E-Learning. 2016; 19(2):129-146.

Pleasant M, 2018. Improving dementia care through online training programs: A systematic review and evaluation. 2017. Graduate Theses and Dissertations. Available at: http://scholarcommons.usf.edu/etd/6745

Pleasant M, Molinari V, Hobday J, et al. An evaluation of the CARES® dementia basics program among caregivers. International Psychogeriatrics. 2017; 29(1):45-56.

Pulman A, Galvin K, Hutchings M, et al. Empathy and dignity through technology: Using lifeworld-led multimedia to enhance learning about the head, heart and hand. Electronic Journal of e-Learning. 2012; 10(3):349-360.

Robertshaw D & Babicova I. Discovering the memory thief: MOOC participants' personal experiences of dementia. Nurse Education in Practice. 2019; 42:102693.

Robertshaw D & Cross A. Experiences of Integrated Care for Dementia from Family and Carer Perspectives: A Framework Analysis of Massive Open Online Course Discussion Board Posts. Dementia. 2019a; 18(4):1492-1506.

Robertshaw D & Cross A. Roles and responsibilities in integrated care for dementia. Journal of Integrated Care. 2019b; **27**(2): 131-140.

Sehgal M, Syed Q, Callahan K, et al. Introducing Aquifer Geriatrics, the American Geriatrics Society National Online Curriculum. Journal of the American Geriatrics Society. 2019; 67(4): 811-817.

Surr C, Sass C, Drury M, et al. A collective case study of the features of impactful dementia training for care home staff. BMC Geriatrics. 2019; 19(1):175.

Tomaz J, van der Molen, Mamede S. The design and program evaluation of a distributed PBL curriculum for training family doctors in Brazil. European Journal of Open, Distance and E-Learning. 2013; 16(1):11-26.

Van Mierlo L, Meiland F, Van de Ven P, et al. Evaluation of DEM-DISC, customized e-advice on health and social support services for informal carers and case managers of people with dementia; a cluster randomized trial. International Psychogeriatrics. 2015; 27(8):1365-1378.

Waldorff F, Steenstrup A, Nielsen B, et al. Diffusion of an e-learning programme among Danish general practitioners: A nation-wide prospective survey. BMC Family Practice. 2008; 9:24.
