# Supplementary material for: The characteristics of effective technology-enabled dementia education: a systematic review and mixed research synthesis
Source: Syst Rev. 2022 Feb 23;11:34. doi: 10.1186/s13643-021-01866-4 (PMC8865181; doi:10.1186/s13643-021-01866-4)
Supplement: Supplementary file 6 — Additional file 6. [file 13643_2021_1866_MOESM6_ESM.docx]

**Additional File 6a. Structured summary of qualitative studies**

| **Citation,**  **Country** | **Context** | **Research Objective** | **Participants** | **Data Collection Methods** | **Findings (Themes)** | **Methodological quality** |
| --- | --- | --- | --- | --- | --- | --- |
| Bentley, Kerr et al. (60),  Australia | IMGs and practice nurses from primary care participated in an online dementia education program | To discuss experiences of working with people with dementia | IMGs (n=13)  and practice nurses (n=11) | Semi-structured interviews were conducted prior to and 1 months after completing the online program | Themes not explicitly reported. | \| Y \| ? \| ? \| ? \| Y \| \| --- \| --- \| --- \| --- \| --- \|   MMAT1 |
| De Witt Jansen, Brazil et al. (61),  UK | Physicians, nurses, and health care assistants from various health care settings participated in ECHO tele-mentoring clinics for assessing and managing pain for people with advanced dementia nearing end of life | To establish reasons for participation in ECHO tele-mentoring clinics; perceptions of the efficacy of the curriculum in addressing learning needs; the application of learning gained to patient care; the impact of participation in clinics on participants’ clinical teams; how, when, and if participants shared knowledge and skills from clinics with others; participants’ perceptions of future pain clinics | *Focus group 1*  Dementia nurse (n=1) Hospice nurse (n=2)  *Focus group 2*  GP (n=1)  Consultant geriatrician (n=1)  Hospice nurse (n=2) | Two focus groups discussion were held upon completion of the final ECHO tele-mentoring clinic | - Knowledge and skills development and dissemination - Protected time - Areas for improvement - The future of ECHO | \| Y \| Y \| Y \| Y \| Y \| \| --- \| --- \| --- \| --- \| --- \|   MMAT1 |
| Jones, Moyle (62)  Australia | Registered nurses, enrolled nurses, personal care workers and diversional therapists from long-term care and nursing students participated in an e-learning intervention (based on the sexualities and dementia education resource for health professionals) | To establish participants’ views of, and their approach and response to, case scenarios on sexuality and dementia. In addition, the ease of use, quality, and effectiveness of the e-learning education resource were evaluated | Nine participants were interviewed | Semi-structured interview (think aloud technique) | - Being happy and well - Conferring with family - Workplace policy - Evaluation self-directed e-learning resource | \| Y \| ? \| ? \| Y \| Y \| \| --- \| --- \| --- \| --- \| --- \|   MMAT1 |
| Kimzey, Mastel-Smith et al. (63)  USA | Nursing students participated in different educational experiences including AD clinical experience, an AD online module, or in a control group. | To establish thoughts and feelings about AD and AD knowledge | Nursing students (n=11)^1^ | Focus group | - Basic AD knowledge - Need for AD education - Negative feelings related to behaviours - Encouraging implications for practice | \| Y \| Y \| Y \| Y \| Y \| \| --- \| --- \| --- \| --- \| --- \|   MMAT1 |

IMG (International Medical Graduate); AD (Alzheimer’s Disease)

1 One student had participated in an AD clinical experience; two students had participated in an AD online module; the remaining 8 participants were from a control group.

**Additional File 6b. Qualitative codes and themes**

| **Pre-TEDE Perceptions on Dementia Education & Training** | | | | | |
| --- | --- | --- | --- | --- | --- |
| **Initial Code** | **Frequency^1^** | **Sources^2^** | | | **Theme** |
| Having varied experience | 3 | Bentley |  |  | Existing strengths and experience |
| Acknowledging previous knowledge sources | 2 | Kimzey |  |  |  |
| Having basic knowledge | 1 | Kimzey |  |  |  |
| Having basic caregiving competencies | 1 | Kimzey |  |  |  |
| Coping with behavioural symptoms | 1 | Kimzey |  |  |  |
| Acknowledging demographic variations | 1 | Bentley |  |  |  |
| Having negative perceptions | 2 | Kimzey |  |  | Knowledge gaps and uncertainty |
| Having knowledge gaps | 2 | Bentley | Kimzey |  |  |
| Need for practical experience | 1 | Kimzey |  |  |  |
| Having a lack of confidence | 1 | Kimzey |  |  |  |
|  | | | | | |
| **Post-TEDE Perceptions on Dementia Education & Training** | | | | | |
| **Initial Code** | **Frequency^1^** | **Sources^2^** | | | **Theme** |
| Developing new knowledge | 6 | Bentley | DeWitt | Jones | Developing core competence and expertise |
| Having the confidence to apply new skills | 4 | Bentley |  |  |  |
| Sharing knowledge | 3 | Bentley | DeWitt |  |  |
| Developing new skills | 3 | Jones | Bentley | DeWitt |  |
| Application of behaviour change in practice | 3 | Bentley | DeWitt |  |  |
| Changing attitudes | 2 | Bentley | Jones |  |  |
| Becoming an expert | 1 | Bentley |  |  |  |
| Developing confidence | 1 | Bentley |  |  |  |
| Handling complexity | 1 | Jones |  |  |  |
| Having access to specialists / experts | 5 | DeWitt |  |  | Involving relevant others in TEDE |
| Benefits of multidisciplinary learning | 3 | DeWitt |  |  |  |
| Learning in groups – benefits | 3 | DeWitt |  |  |  |
| Building a sense of community | 2 | DeWitt |  |  |  |
| Reassurance of existing practices | 2 | DeWitt |  |  |  |
| Incorporating family perspectives | 2 | Jones |  |  |  |
| Learning in groups – challenges | 1 | DeWitt |  |  |  |
| Incorporating different perspectives | 1 | DeWitt |  |  |  |
| Managing isolation | 1 | Jones |  |  |  |
| Protecting learning time | 4 | DeWitt |  |  | Optimising feasibility |
| Forward planning | 4 | DeWitt |  |  |  |
| Acknowledging convenience and flexibility | 3 | DeWitt | Jones |  |  |
| Dealing with technical issues | 2 | DeWitt |  |  |  |
| Incorporating guidance / Models of care | 1 | Jones |  |  |  |

1 Number of times that the initial code was identified in the source data

2 First author only – citations provided in the structured summary of qualitative studies.
